# Deep learning model integrating cfDNA methylation and fragment size profiles for lung cancer diagnosis

**DOI:** 10.1038/s41598-024-63411-2

**Published:** 2024-06-26

**Authors:** Minjung Kim, Juntae Park, Seonghee Oh, Byeong-Ho Jeong, Yuree Byun, Sun Hye Shin, Yunjoo Im, Jong Ho Cho, Eun-Hae Cho

**Affiliations:** 1 Genome Research Center, GC Genome, Yongin-si, Korea; 2 Division of Pulmonary and Critical Care Medicine, Department of Medicine, Samsung Medical Center, Sungkyunkwan University School of Medicine, Seoul, Korea; 3 Smart Healthcare Research Institute, Research Institute for Future Medicine, Samsung Medical Center, Seoul, Korea; 4 Department of Thoracic and Cardiovascular Surgery, Samsung Medical Center, Sungkyunkwan University School of Medicine, Seoul, Korea

**Keywords:** Lung cancer, ctDNA, Methylation, MeDIP-seq, EM-seq, Next-generation sequencing, Cancer screening, Lung cancer, Methylation analysis, DNA methylation

## Abstract

Detecting aberrant cell-free DNA (cfDNA) methylation is a promising strategy for lung cancer diagnosis. In this study, our aim is to identify methylation markers to distinguish patients with lung cancer from healthy individuals. Additionally, we sought to develop a deep learning model incorporating cfDNA methylation and fragment size profiles. To achieve this, we utilized methylation data collected from The Cancer Genome Atlas and Gene Expression Omnibus databases. Then we generated methylated DNA immunoprecipitation sequencing and genome-wide Enzymatic Methyl-seq (EM-seq) form lung cancer tissue and plasma. Using these data, we selected 366 methylation markers. A targeted EM-seq panel was designed using the selected markers, and 142 lung cancer and 56 healthy samples were produced with the panel. Additionally, cfDNA samples from healthy individuals and lung cancer patients were diluted to evaluate sensitivity. Its lung cancer detection performance reached an accuracy of 81.5% and an area under the receiver operating characteristic curve of 0.87. In the serial dilution experiment, we achieved tumor fraction detection of 1% at 98% specificity and 0.1% at 80% specificity. In conclusion, we successfully developed and validated a combination of methylation panel and a deep learning model that can distinguish between patients with lung cancer and healthy individuals.

## Introduction

Lung cancer is the second most common cancer worldwide (11.4%) and a significant contributor to cancer-related deaths (18%)^1^. The early detection of lung cancer is crucial for improving patient survival rates^2^. Lung cancer screening using low-dose computed tomography has effectively reduced mortality^3^. However, it has limitations, including a high false-positive rate, risk of radiation exposure, and cost concerns^4^. Consequently, there is a growing need for non-invasive and accurate diagnostic approaches to detect lung cancer at an early stage.

Cell-free DNA (cfDNA) has emerged as a promising method for early non-invasive cancer diagnosis^5,6^. Circulating tumor DNA (ctDNA) is released from tumor cells^7^ and reflects tumor-specific methylation changes^8^. Additionally, ctDNA fragments are shorter than normal cfDNA fragments^9^. Considering their characteristics, numerous studies involving ctDNAs have been conducted for cancer diagnosis^8–10^.

The changes DNA methylation manifest predominantly during the initial stages of tumor development^11^, and the identification of modified DNA methylation patterns has demonstrated efficacy in the early detection of cancer^12,13^. Recent studies have identified DNA methylation markers for lung cancer detection^14,15^. Furthermore, to improve sensitivity, ongoing studies have employed these methylation markers to create targeted panels and harness methylation data at a high-depth level^16–18^.

Various analysis methods using fragment size as another feature for early diagnosis of cancer have been studied^5,10,19^. These studies were developed based on the knowledge that ctDNA is shorter than normal cfDNA. Previous studies have identified differences in cfDNA fragmentation profiles between patients with lung cancer and healthy individuals, and a cfDNA fragmentation profile analysis method called DELFI was developed to increase the sensitivity of early cancer detection^5^. In another study, a model was built using not only the ratio of short and long fragments used in DELFI, but also fragment size distribution and fragment coverage^10^. A recent study developed a cancer diagnostic model by integrating fragment size and read distribution falling across nucleosome-depleted regions (NDRs)^19^. Consequently, the excellence of each feature of fragment size and methylation information has been verified, but the correlation between methylation and fragment size has not been studied.

In this study, we postulated that augmenting information on DNA methylation patterns with fragment size profiles could enhance the accuracy and sensitivity of lung cancer detection. Our primary objective was to develop a deep learning algorithm for lung cancer diagnosis using cfDNA methylation and the fragment size profile (MFS). We initially determined the distinct methylation markers between patients with lung cancer and healthy individuals. Based on these markers, a targeted Enzymatic Methyl-seq (EM-seq) panel was designed. Subsequently, we harnessed data from this targeted EM-seq panel to scrutinize the MFS of lung cancer and healthy samples at high depths, and these profiles served as the foundation for our algorithm development.

## Method

### Participants

The Institutional Review Board (IRB No. SMC 2022-05-027 for patient samples; GCL-2017-1008-03, GCL-2020-1002-06, GCL-2021-1049-07 for healthy samples) approved the collection of plasma and tissues from patients with lung cancer and healthy individuals. Informed consent was obtained from all participants. All research was conducted in accordance with relevant guidelines and regulations. Patients with lung cancer were diagnosed histologically and treatment naive. The clinical data from the methylated DNA immunoprecipitation sequencing (MeDIP-seq), Whole-genome Enzymatic Methyl-seq (WGEM-seq), Twist Human Methylome Panel, and targeted EM-seq panel are summarized in Supplementary Tables 1, 2, 3 and Table 1. Details of the dataset composition are provided in Supplementary Table 4.

**Table 1:** Clinical data in targeted EM-seq panel.

	Training/validation set		Test set	
	Lung caner	healthy	Lung caner	healthy
Total, n (%)	102	42	40	14
Age, years				
Mean	64	62	63	42
Range	44–82	52–86	32–81	30–65
Gender				
Male, n (%)	67 (65.69)	6 (14.29)	28 (70.00)	6 (42.86)
Female, n (%)	35 (34.31)	36 (85.71)	12 (30.00)	8 (57.14)
Stage				
I, n (%)	31 (30.39)	–	7 (17.50)	–
II, n (%)	9 (8.82)	–	7 (17.50)	–
III, n (%)	38 (37.25)	–	21 (52.50)	–
IV, n (%)	15 (14.71)	–	3 (7.50)	–
Unknown, n (%)	9 (8.82)	–	2 (5.00)	–
Subtype				
ADC, n (%)	61 (59.80)	–	20 (50.00)	–
NSCLC-NOS, n (%)	2 (1.96)	–	7 (17.50)	–
SCC, n (%)	28 (27.45)	–	8 (20.00)	–
SCLC, n (%)	4 (3.92)	–	3 (7.50)	–
Unknown, n (%)	7 (6.86)	–	2 (5.00)	–

*ADC* adenocarcinoma, *NSCLC-NOS* non-small cell lung cancer-not otherwise specified, *SCC* squamous cell carcinoma, *SCLC* small cell lung cancer.

### DNA extraction

The peripheral blood collected in Streck tubes (Streck, USA) underwent a two-step centrifugation protocol for the separation of plasma and Buffy coat. After centrifugation at 3000 rpm for 10 min at 25 °C, a second centrifugation was performed at 16,000×*g* for 10 min at 25 °C.

The plasma was separated and cfDNA was extracted using a specific kit. For MeDIP-seq, cfDNA was automatically extracted using the chemagic DNA Blood200 kit (PerkinElmer, USA) through the chemagic MSM I instrument (PerkinElmer). Twist Human Methylome Panel and targeted EM-seq panel utilized the Mag-Bind cfDNA kit (Omega Bio-Tek, USA) for manual extraction. In the cfDNA extraction, 2 ml of plasma was utilized, with an elution volume of 50 µl.

Genomic DNA (gDNA) extraction was performed on both buffy coat and fresh frozen tissue samples. For fresh frozen tissue samples, 10–30 ng of tissue was homogenized using the Homogenizer FastPrep-24 system (MP Biomedicals, USA). Subsequently, for WGEM-seq, gDNA was extracted using the QIAmp DNA Mini kit (Qiagen, Germany) from separated buffy coat and fresh frozen tissue samples.

### Serial sample preparation to determine the limit of detection (LOD)

We conducted limit of detection experiments using plasma samples from a cancer patient and a healthy individual, diluted to specific tumor fraction ratios. For a lung cancer sample, a tumor fraction of 15% was predicted using ichorCNA (v0.2.0)^20^. The tumor fraction range was set across five levels: undiluted (tumor fraction 15%), 1%, 0.5%, 0.1%, and 0% (representing a healthy individual).

### MeDIP-seq

The extracted cfDNA (10 ng) was prepared into libraries using the TruSeq Nano DNA HT Library Prep Kit (Illumina, USA). Following the adapter ligation step, a 5mC immunoprecipitation was conducted using the iPure Kit V2 (Diagenode, USA) at 10 rpm and 4 °C for 17 h, followed by PCR amplification for 13 cycles. The concentration and size distribution of the resulting libraries were measured using the Qubit dsDNA HS Assay Kit (Invitrogen, USA) and TapeStation 4200 (Agilent Technologies, USA). The prepared libraries were sequenced on the NovaSeq 6000 sequencer (Illumina) in 150-bp paired-end mode, generating approximately 100 million reads per sample.

Adapter trimming and quality trimming of fastq files were performed using Trim Galore (version 0.6.6)^21^. Nucleotide fragments were aligned to the human reference genome (hg19) using the BWA Alignment Tool (v0.7.17-r1188). Duplicate PCR fragments were removed, and fragments with a mapping quality below 10 were excluded using SAMtools (v1.11). Chromosomes 1–22 were retained, while the others were discarded. We divided the entire genome into 300 bp bins and calculated the read counts for each bin, excluding the regions in the blacklist^22^. And bins with a total read count of 10 or less across all samples were excluded. Normalization of the 300-bp bins was performed using the trimmed mean of M-values (TMM)^23^ with the edgeR (v3.28.1) R package^24^.

### WGEM-seq

The gDNA (200 ng) was fragmented to sizes ranging from 240 to 290 bp using Covaris instrument (Covaris, USA). The library was prepared using the NEBNext enzymatic methyl-seq kit (New England Biolabs, USA) with 200 ng of DNA. The library preparation involved a methylation conversion step, wherein ten-eleven translocation dioxygenase 2 (TET2) and APOBEC enzymes were employed to replace non-methylated cytosines with Uracil. The final DNA library's size and concentration were determined using the Qubit dsDNA HS Assay Kit (Invitrogen) and TapeStation 4200 (Agilent Technologies). In the last step, the prepared DNA libraries were sequenced on the NovaSeq 6000 sequencer (Illumina) in 150-bp paired-end mode, generating approximately 600 million reads per sample.

We performed adapter and quality trimming of FASTQ files using Trim Galore. The nucleotide fragments were then aligned to the hg19 reference genome using Bismark tool (v0.23.0)^25^, and duplicate PCR fragments were removed using the deduplicate_bismark. We used SAMtools view to exclude nucleotide fragments with a mapping quality of less than 10 and restricted them to chromosomes 1 to 22. Methylation calling was performed using the Bismark_methylation_extractor. Beta values were calculated using the methylKit R package (v1.12.0)^26^ to quantify the methylation levels. The beta values were obtained from CpG sites with a minimum depth of 5 or more.

### Twist Human Methylome Panel and targeted EM-seq panel

The Twist Human Methylome Panel (Twist Bioscience, USA) targets biologically relevant methylation markers across 123 Mb of genomic content, encompassing 3.98M CpG sites. Our custom-designed Targeted EM-seq panel comprises 366 lung cancer-specific methylation markers that differentiate normal samples from cancer samples. Manufactured by Twist Bioscience, this panel spans 0.1 Mb and includes 5K CpG sites.

Prepared DNA libraries using the NEBNext enzymatic methyl-seq kit (New England Biolabs), utilizing 2–100 ng of extracted cfDNA. Methylation conversion involved replacing unmethylated cytosines with uracil through TET2 and APOBEC enzymes. Eight sample groups were created by combining 200 ng from each library for hybridization. Subsequently, the process focused on capturing the specific target from the hybridized sample. The concentration and size distribution of the resulting libraries and captured DNA were measured using the Qubit dsDNA HS Assay Kit (Invitrogen) and TapeStation 4200 (Agilent Technologies). Sequencing was performed on the NovaSeq 6000 and MiSeq Dx sequencers (Illumina) in 150-bp paired-end mode, Twist Human Methylome Panel and targeted EM-seq panel achieved average sequencing depths of 220× and 700× per sample. Data preprocessing was performed in the same way as described for WGEM-seq. Beta values were obtained for CpG sites with a minimum coverage of 10 and 20 for the Twist Human Methylome panel and Targeted EM-seq panel, respectively.

### Methylation markers on the Infinium HumanMethylation450 (450K) BeadChip array and MeDIP-seq

The Infinium HumanMethylation450 (450K) BeadChip array data with the title beginning as GDC TCGA was obtained from the University of California Santa Cruz (UCSC) Xena database (https://xenabrowser.net/datapages/). The data consists of 458 primary solid tumor samples and 32 adjacent normal tissue samples for lung adenocarcinoma (ADC), as well as 370 primary solid tumor samples and 42 adjacent normal tissue samples for lung squamous cell carcinoma (SCC). Additionally, the 450K array data of 656 normal blood samples were obtained from Gene Expression Omnibus (GEO) databases (https://www.ncbi.nlm.nih.gov/geo/; GSE40279)^27^. We obtained beta values for each CpG site from the 450K array data and excluded CpG sites with missing values. The dataset was divided into a discovery set and a validation set, and markers were selected using the discovery set and verified using the validation set (Supplementary Table 4a,b). Differentially methylated regions (DMRs) were selected, regions that exhibited differences between lung cancer tissues and adjacent normal tissues, as well as differences between lung cancer tissues and normal blood samples. This selection was made using the Limma (v3.46.0) R package^28^, regions where the false discovery rate (FDR, Benjamini–Hochberg method) was < 0.01 and the absolute delta beta was > 0.25.

MeDIP-seq data was generated in-house from 25 patients with lung cancer and 190 healthy individuals. MeDIP-seq also divided the dataset into a discovery set and a validation set, the same as the 450K array data (Supplementary Table 4c). DMRs were identified between lung cancer and healthy samples. Using the edgeR R package, we selected a region with an FDR (Benjamini–Hochberg method) value of less than 0.05 and extracted CpGs within the region.

### Methylation markers on the 450K array and WGEM-seq

The 450K array was processed following the same protocol outlined in the section “Methylation markers on the Infinium HumanMethylation450 (450K) BeadChip array and MeDIP-seq”. In WGEM-seq data, We identified DMRs by comparing methylation patterns between seven lung cancer tissues and seven adjacent normal tissues, and between seven lung cancer tissues and ten normal white blood cells (WBC) (Supplementary Table 4d). The filtering criteria included an absolute difference > 25 and a q-value < 0.01, calculated using the methylKit R package. P-values were calculated using logistic regression and adjusted to q-values using the SLIM method^29^.

### Significant methylation markers in the cfDNA

We utilized Twist Human Methylome Panel data from five patients with lung cancer and seven healthy individuals (Supplementary Table 4e). Three steps were performed to perform further filtering on the selected CpGs.

First, the beta value was used to calculate the area under the receiver operating characteristic curve (AUC) with Scikit-learn Python library (v1.0.2)^30^ to distinguish lung cancer samples from healthy samples. Regions with AUC values above 0.65 were considered significant. Second, we included regions where the absolute difference between lung cancer and healthy samples was greater than 3 and the absolute q-value was less than 0.05 using the methylKit R package. Finally, we selected regions where the standard deviation of healthy samples was less than 0.05 using the R software (v3.6.3).

### MFS in cfDNA

To generate MFS inputs, we utilized targeted EM-seq panel data. The targeted EM-seq panel comprises cfDNA data from 142 lung cancer patients and 56 healthy individuals. We conducted 100-bp binning based on genomic coordinates and selected bins with three or more methylation markers. Next, methylation levels were measured based on fragment sizes ranging from 120 to 220 in 10-bp intervals.

Methylation level = number of methylated cytosines/total cytosines, where total cytosines ≥ 20.

The total cytosines is the sum of the number of methylated cytosines and the number of unmethylated cytosines. The created MFS table is a 2D table, where the x-axis is the genomic position, the y-axis is the fragment size, and the value is the methylation level (Supplementary Fig. 1). For regions with total cytosines < 20, missing values were imputed using the median methylation level for that fragment size.

### Deep learning model generation

Model development employed MFS generated from the targeted EM-seq panel of 142 lung cancer patients and 56 healthy individuals as input. We developed a convolutional neural network (CNN) model to discriminate between healthy individuals and patients with lung cancer using a 2D vector MFS table as input data. The dataset was preprocessed by applying standardization scaling using healthy samples from the training and validation sets. We divided the entire dataset into training, validation, and test sets (Table 1). The training set was used for model training, the validation set for hyper-parameter tuning, and the test set for evaluating the final model performance. Hyperparameter tuning is the process of optimizing the values of various parameters (number of convolution layers, number of dense layers, number of convolution filters, etc.) that make up a CNN model. Bayesian optimization technique is used in the hyperparameter tuning process. When the validation loss starts to increase compared to the training loss, the model is considered to be overfitting and the model training is stopped. The performance of multiple models obtained through hyperparameter tuning is compared using the validation set. The model with the best performance on the validation set is selected as the optimal model, and the final performance is evaluated using the test set. Given a 2D vector MFS table of a specific sample, the trained CNN model calculated the probability that it was a healthy individual or a patient with lung cancer. The sigmoid function in the final layer was used for calculation. Patients with lung cancer and healthy individuals were classified based on a predicted probability of 0.5.

### Model construction utilizing fragment size and methylation level feature

To compare the performance with the MFS feature, we used cfDNA data from the targeted EM-seq panel of 142 lung cancer patients and 56 healthy individuals. For fragment size features, we used the DELFI^5^ method to calculate the ratio of short fragments. The ratio of short fragments was calculated by dividing the number of short fragments (100–150 bp) by the number of long fragments (151–220 bp) for each 100 bp bin. As for the methylation level feature, we quantified methylation levels in 100 bp bins following the same protocol described in the “MFS in cfDNA” section, but the data was integrated without being divided by fragment size. Standardization scaling was applied to both computed features, utilizing healthy samples from the training and validation sets. Subsequently, a CNN model was trained using these features.

### Statistical analyses

Methylation markers were selected using the Limma R package for the 450K array, edgeR R package for MeDIP-seq, and methylKit R package for WGEM-seq. Marker filtering used Scikit-learn Python library, methylKit R package, and R software. To evaluate the model performance, we utilized metrics including the area under the receiver operating characteristic curve (AUROC), accuracy, and sensitivity values fixed at 80%, 95%, and 98% specificity. All evaluation metrics were performed using a custom Python script (v3.8.1), with 95% confidence intervals (CI) obtained from 2000 bootstrap iterations.

## Results

### Overview of marker selection and test

**Figure 1:**
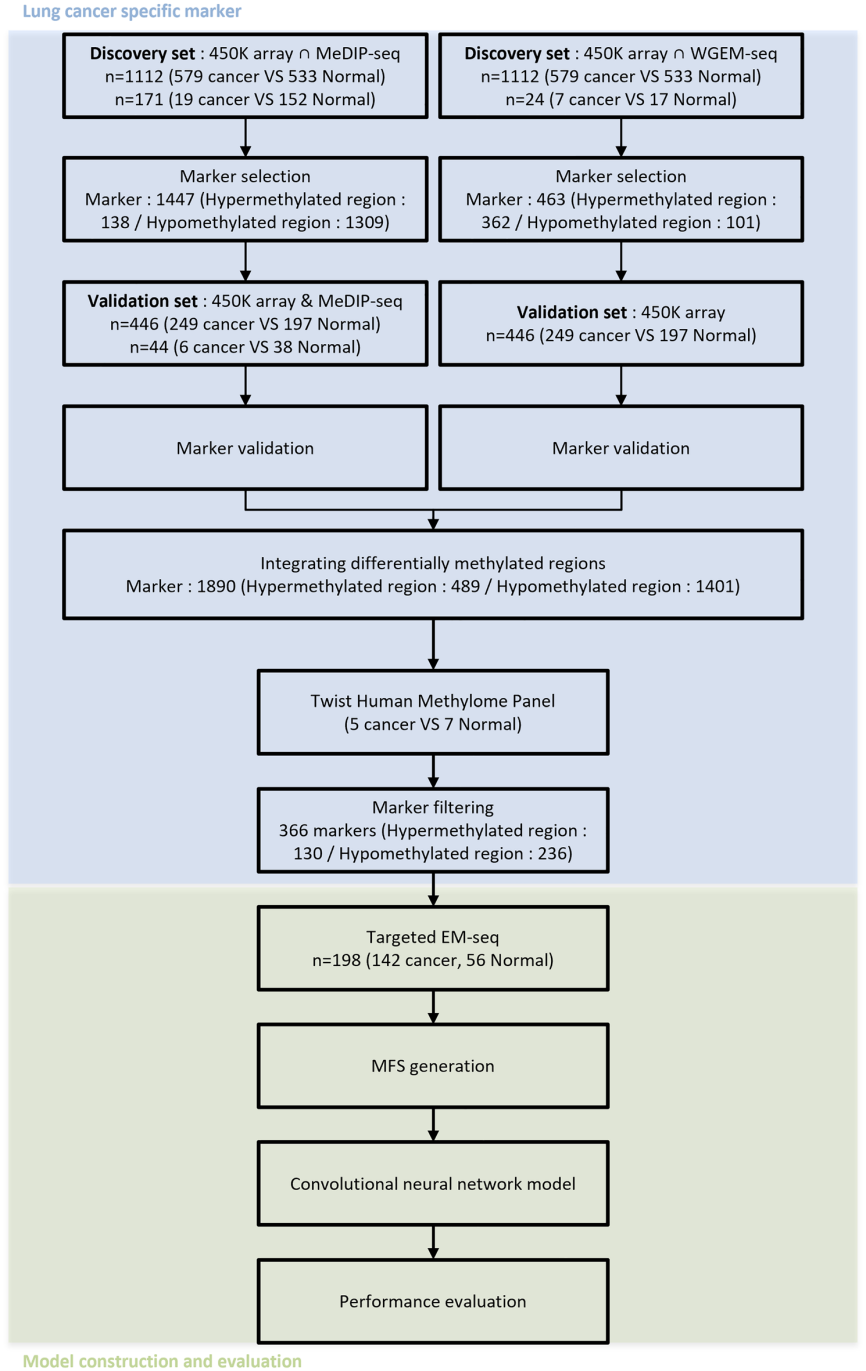
Study workflow. We selected a lung cancer specific methylation marker and designed a targeted EM-seq panel using the marker. We developed a deep learning model integrating methylation and fragment size (MFS) using targeted EM-seq panel data, and evaluated model performance.

To identify the methylation markers for classifying lung cancer and healthy samples, we collected the 450K array data from databases. We then generated new data in-house using MeDIP-seq, WGEM-seq, and the Twist Human Methylome Panel. The 450K array data included 828 primary solid tumor samples, 74 adjacent normal tissue samples, and 656 normal blood samples. For MeDIP-seq, 25 lung cancer samples and 190 normal samples were used. EM-seq was performed on seven lung cancer tissue samples, seven paired adjacent normal tissue samples, and ten normal WBCs (Fig. 1). From the discovery sets of the 450K array and MeDIP-seq, as well as the 450K array and WGEM-seq, we selected 1447 and 463 differentially methylated markers, respectively. Subsequently, we validated whether the selected markers showed differences between cancer and normal samples in the validation set. Subsequently, the markers were integrated, resulting in a total of 1890 markers. Additionally, we conducted further marker filtering using data from 5 lung cancer samples and 7 healthy samples from the Twist Human Methylome Panel.

A targeted EM-seq panel was designed using the selected markers. This panel generated data from 142 lung cancer patients and 56 healthy individuals. The data was used to create an MFS table, which was then used as input to train a CNN model. The performance of the trained model was evaluated.

### Differential methylated regions between patients with lung cancer and healthy samples

Using the 450K array data, we identified DMRs to primary solid tumors in ADC and SCC. Specifically, we compared 320 ADC and 259 SCC primary solid tumor samples with 32 and 42 adjacent normal tissues, respectively, along with 459 normal blood samples. We selected DMRs that were distinct in primary lung tumors and adjacent normal tissues as well as those that were differentially methylated between lung tissues and normal blood samples. And using MeDIP-seq data, we identified DMRs specific to lung cancer by comparing 19 lung cancer samples with 152 healthy samples. We identified 138 hypermethylated and 1309 hypomethylated lung cancer-specific markers in the two datasets (Supplementary Table 5, Supplementary Fig. 2). When we identified these markers on the validation sets of the 450K array and MeDIP-seq using t-SNE plots, differences between cancer and normal samples were evident. (Supplementary Fig. 3a).

Using EM-seq data, we identified DMRs that were specific to lung cancer by comparing seven lung cancer tissues with their paired adjacent normal tissues, as well as seven lung cancer tissues with ten normal WBCs. We selected 362 hypermethylated and 101 hypomethylated markers in the EM-seq and the 450K array datasets (Supplementary Table 5, Supplementary Fig. 2). When we analyzed t-SNE plots using these markers on the validation set of the 450K array and the full data set from WGEM-seq, we identified differences between cancer and normal samples (Supplementary Fig. 3b).

Using the 450K array data, we identified subtype-specific DMRs and common DMRs in ADC and SCC. In hypermethylated regions, subtype-specific DMRs were found to be 16.9% for ADC and 52.8% for SCC, while common DMRs accounted for 30.3% (Supplementary Table 6, Supplementary Fig. 4). In hypomethylated regions, subtype-specific DMRs were 2.3% for ADC and 92.6% for SCC, with common DMRs representing 5.2%. Due to the relatively low proportion of common DMRs, we included subtype-specific DMRs when integrating DMRs (Supplementary Fig. 5).

### Significant methylated markers

In this study, we selected lung cancer-specific methylated markers from three different sources: the 450K array, MeDIP-seq, and EM-seq. We included markers found in both the 450K array and MeDIP-seq as well as those in both the 450K array and WGEM-seq. Subsequently, we conducted marker filtering using high-depth Twist Human Methylome Panel data. The samples used were 5 lung cancer patients and 7 healthy individuals. After methylation marker filtering, we identified 130 hypermethylated and 236 hypomethylated markers. Examination of these markers in the Twist Human Methylome Panel data using t-SNE plot and heatmaps clearly revealed differences between cancer and healthy samples (Supplementary Fig. 6). The hypermethylated markers were predominantly located in the introns (34.2%), exons (23.4%), and intergenic regions (16.5%), followed by promoters (13.3%) (Supplementary Fig. 7a). The hypomethylated markers were primarily found in introns (38.3%), intergenic regions (32.4%), and exons (14.1%) (Supplementary Fig. 7b). Moreover, when comparing the hypermethylated and hypomethylated markers, the hypermethylated markers were enriched in CpG islands.

### Differences in fragment size and methylation levels between cancer and healthy samples

**Figure 2:**
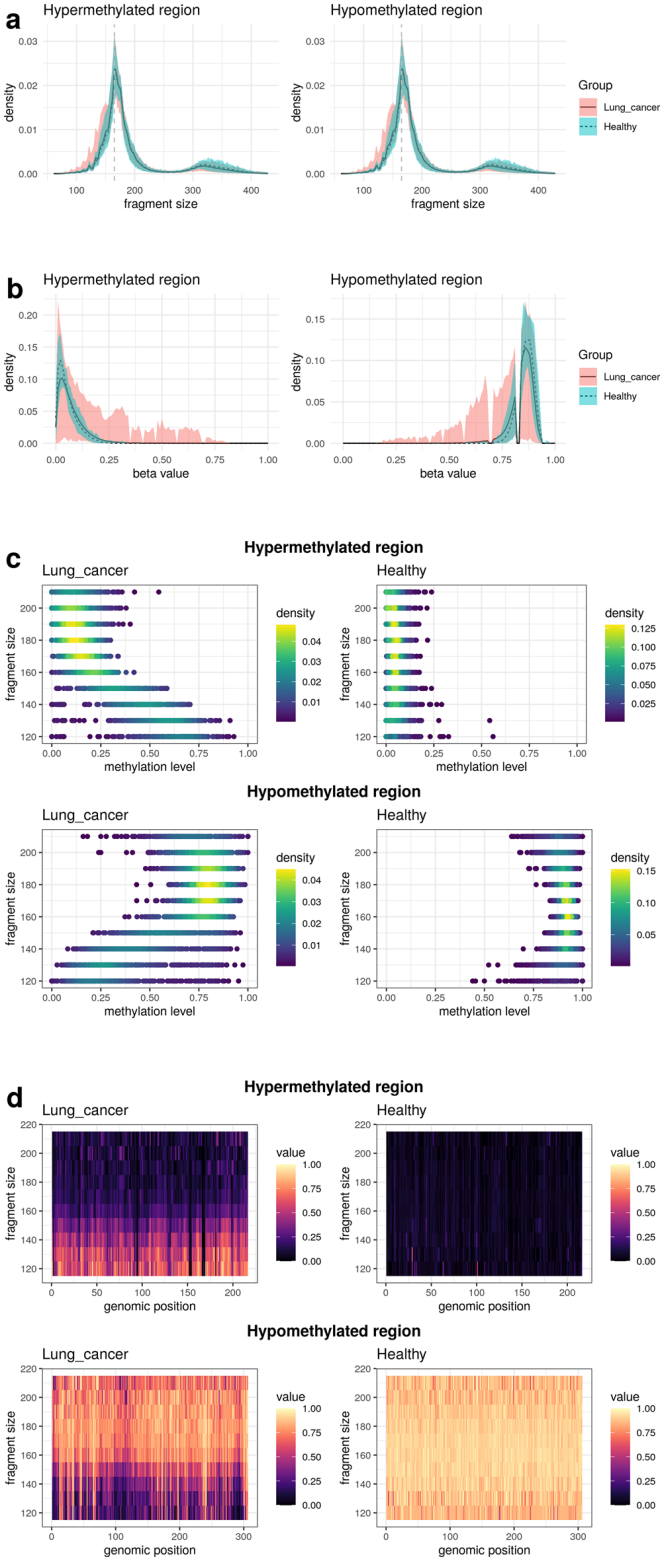
cfDNA methylation and fragment size profiles analysis. (**a**) Distribution of fragment size. (**b**) Distribution of beta value. (**c**) Correlation between fragment size and methylation level. Fragment size (y axes) and methylation level (x axes) dot plot. (**d**) Methylation and fragment size profiles (MFS) of cell-free DNA. The x and y axes represent genomic position (100 bp bin) and fragment size profile respectively. And the value is the methylation level.

When examining the fragment size distribution in the targeted EM-seq panel between patients with cancer and healthy individuals, it was observed that the cancer samples had shorter fragment sizes than the healthy samples (Fig. 2a). To assess the difference between cancer and healthy samples in methylation levels, we compared using beta values. First, we removed noise, and during this process, we confirmed that while noise was removed, the cancer signal remained intact (Supplementary Fig. 8). In terms of methylation levels, patients with cancer exhibited higher methylation levels than the healthy individuals in hypermethylated regions, whereas in hypomethylated regions, patients with cancer exhibited lower methylation levels than their healthy individuals (Fig. 2b). It was confirmed that the methylation pattern in hypermethylated/hypomethylated regions of the Targeted EM-seq dataset was reproduced similarly to that of the dataset used for marker selection.

### Differences in methylation levels depending on fragment size between cancer and healthy samples

We investigated the correlation between fragment size and methylation level. The cfDNA MFS table was used to investigate differences in methylation levels based on fragment size. In the hypermethylated regions in cancer samples, methylation levels tended to increase as fragment size decreased, whereas in healthy samples, methylation levels remained consistently low regardless of fragment size (Fig. 2c). Conversely, in hypomethylated regions, cancer samples exhibited a decrease in methylation levels as the fragment size decreased, whereas healthy samples maintained consistently higher methylation levels irrespective of fragment size.

Moreover, when examining the MFS table, in the hypermethylated regions, cancer samples exhibited higher methylation levels in most regions as the fragment size decreased, in contrast to healthy samples (Fig. 2d). In the hypo-methylated regions, shorter fragment sizes corresponded to lower methylation levels in most regions, distinguishing cancer samples from healthy samples.

### Compare models of different regions and features

**Figure 3:**
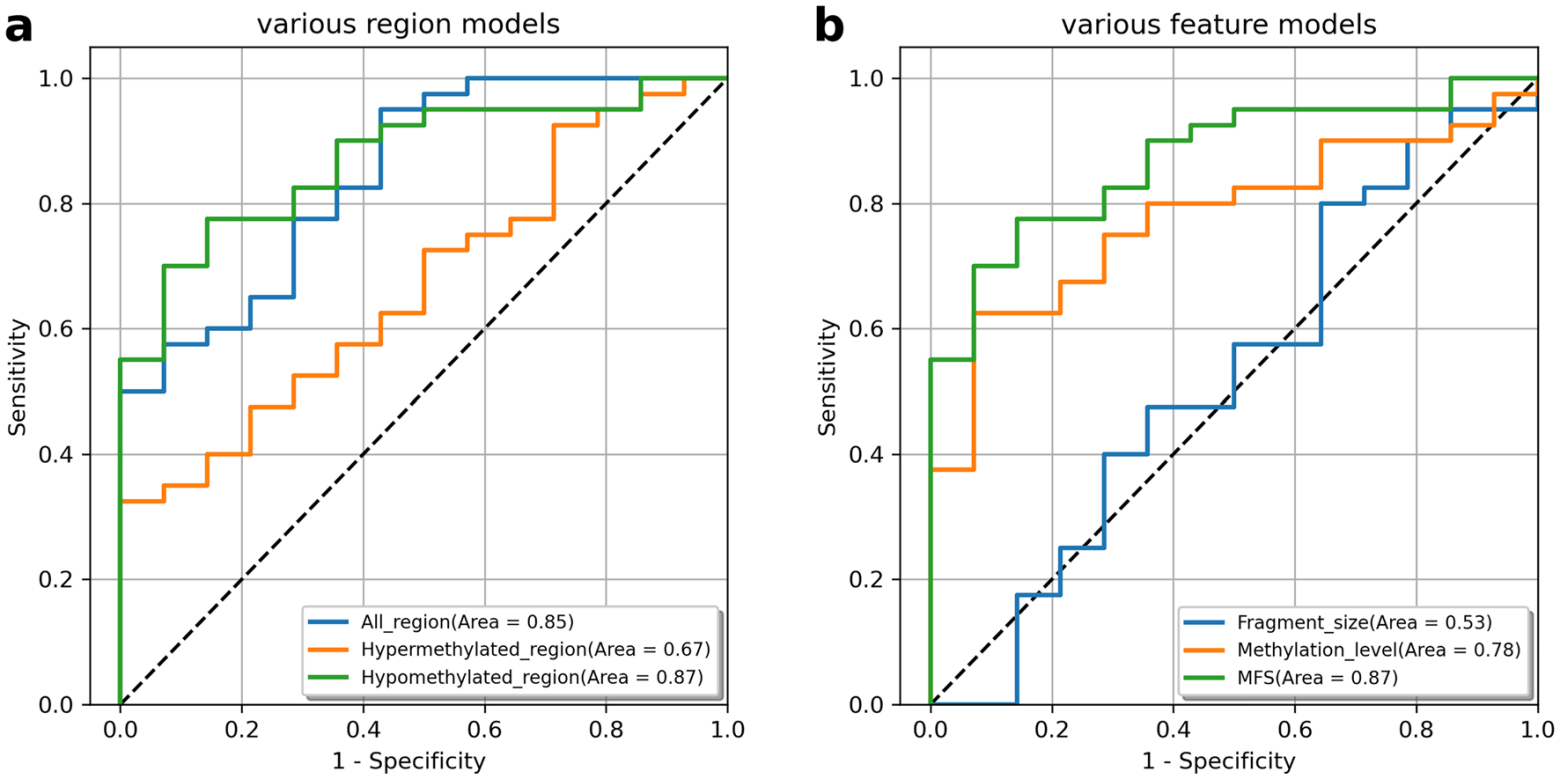
Assessing the performance of models across different regions and features. (**a**) Comparison of the performance of various region models in the test set. (**b**) Comparison of the performance of various feature models in the test set.

We evaluated the model performance on all regions, hypermethylated regions only, and hypomethylated regions only, using the MFS table as an input feature. The test set AUC was 0.85, 0.67, and 0.87, respectively. These results confirm that the hypomethylated region model significantly outperformed the all region and hypermethylated region models (Fig. 3a).

We then compared the performance of MFS with methylation level and fragment size features. Each feature was calculated from hypomethylated regions. On the test set, we found that the performance of methylation level model (AUC = 0.78) was higher than fragment size model (AUC = 0.53), and the performance of MFS model (AUC = 0.87) was higher than methylation level model (AUC = 0.78) (Fig. 3b).

### Performance of the MFS feature

**Figure 4:**
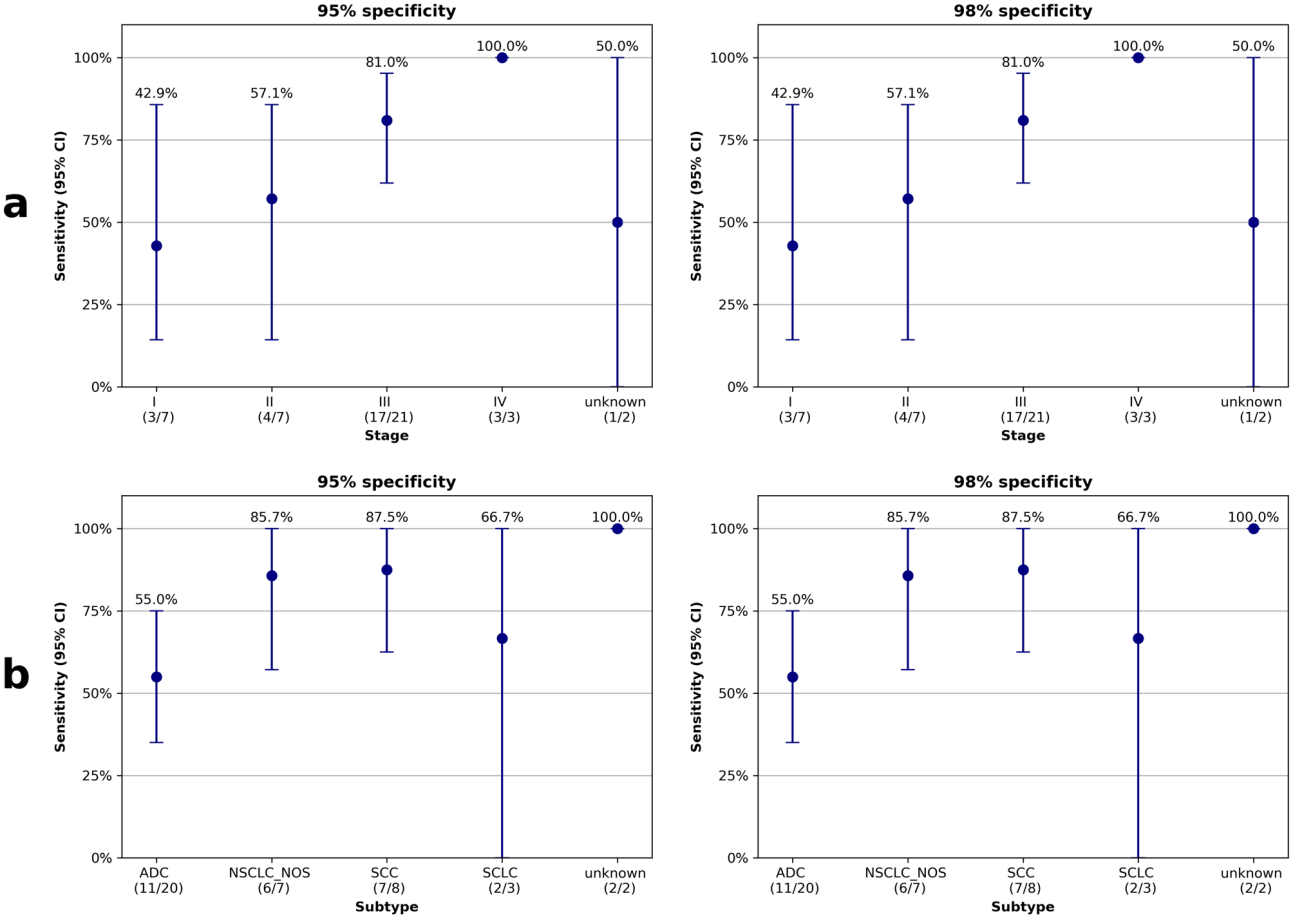
Lung cancer classification performance in the test set. (**a**) Cancer detection sensitivities by stage. (**b**) Cancer detection sensitivities by subtype.

When we evaluated the MFS model of hypomethylated region, the AUC and accuracy for the test set was found to be 0.87 (95% CI: 0.77–0.96) and 81.5% (95% CI: 72.2–90.7%), respectively. At a specificity of 98%, the sensitivity for cancer detection was 70.0% (95% CI: 55.0%–82.5%) (Table 2). The sensitivities for different stages, including stages I, II, III, and IV, at 98% specificity were 42.9% (95% CI: 14.3%–85.7%), 57.1% (95% CI: 14.3%–85.7%), 81.0% (95% CI: 61.9%–95.2%), and 100% (95% CI: 100%–100%), respectively (Fig. 4a, Table 3). Additionally, the sensitivity for distinguishing between ADC and SCC at 98% specificity was 55.0% (95% CI: 35.0%–75.0%) and 87.5% (95% CI: 62.5%–100%), respectively (Fig. 4b, Table 3). Notably, the model performed better in SCC than in ADC.

**Table 2:** The cancer detection performance in the test set.

Model	AUC (95% CI)	Accuracy (%) (95% CI)	Sensitivity at 95% specificity (%) (95% CI)	Sensitivity at 98% specificity (%) (95% CI)
MFS model	0.87 (0.77–0.96)	81.5% (72.2–90.7%)	70.0% (55.0–82.5%)	70.0% (55.0–82.5%)

*CI* confidence interval.

**Table 3:** The cancer detection performance by stage and subtype in the test set.

	Type	AUC (95% CI)	Accuracy (%) (95% CI)	Sensitivity at 95% specificity (%) (95% CI)	Sensitivity at 98% specificity (%) (95% CI)
Stage	I	0.83 (0.62–0.98)	61.9% (42.9–81.0%)	42.9% (14.3–85.7%)	42.9% (14.3–85.7%)
	II	0.78 (0.52–0.97)	61.9% (42.9–81.0%)	57.1% (14.3–85.7%)	57.1% (14.3–85.7%)
	III	0.91 (0.80–0.99)	77.1% (65.7–88.6%)	81.0% (61.9–95.2%)	81.0% (61.9–95.2%)
	IV	1.00 (1.00–1.00)	58.8% (41.2–82.4%)	100.0% (100.0–100.0%)	100.0% (100.0–100.0%)
	Unknown	0.79 (0.50–1.00)	56.2% (31.2–81.2%)	50.0% (0.0–100.0%)	50.0% (0.0–100.0%)
Subtype	ADC	0.80 (0.64–0.93)	70.6% (55.9–85.3%)	55.0% (35.0–75.0%)	55.0% (35.0–75.0%)
	NSCLC-NOS	0.96 (0.86–1.00)	66.7% (52.4–85.7%)	85.7% (57.1–100.0%)	85.7% (57.1–100.0%)
	SCC	0.96 (0.84–1.00)	68.2% (54.5–86.4%)	87.5% (62.5–100.0%)	87.5% (62.5–100.0%)
	SCLC	0.88 (0.62–1.00)	58.8% (41.2–82.4%)	66.7% (0.0–100.0%)	66.7% (0.0–100.0%)
	Unknown	0.96 (0.86–1.00)	56.2% (31.2–81.2%)	100.0% (100.0–100.0%)	100.0% (100.0–100.0%)

To delve into the reasons behind the observed lower performance of ADC compared to SCC, we conducted an analysis of sensitivity by dataset. The findings revealed that the ADC samples were not skewed towards stage I, and the model was not trained on biased data (Supplementary Fig. 9). Our findings align with previous studies that have also reported lower performance of ADC compared to SCC and SCLC^31^.

### Performance of serial dilution

**Figure 5:**
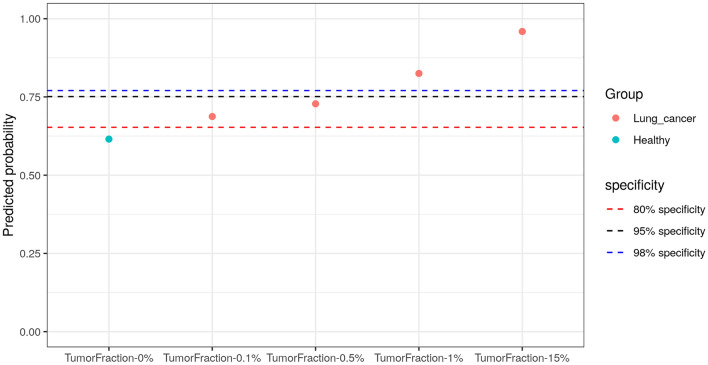
Predicted probability of the serial dilution samples. A lung cancer sample with a tumor fraction of 15% and a healthy sample were serially diluted. The predicted probability was obtained using the trained MFS model.

Serial dilutions were performed using a lung cancer sample with a tumor fraction of 15% and a healthy sample. The dilution results showed a progressively increasing predicted probability as the tumor fraction increased (tumor fraction: 0.1%, 0.5%, 1% and 15%). Detection was achievable at 98% specificity down to 1%, and additionally, we detected low tumor fractions of 0.1% at 80% specificity ﻿(Fig. 5).

## Discussion

In this study, we identified DNA methylation biomarkers for lung cancer diagnosis and developed a CNN model for lung cancer detection using the biomarkers. The selected methylation biomarkers demonstrated differences between lung cancer and normal samples in independent datasets. Moreover, the MFS diagnostic model, which combines cfDNA methylation and fragment size information, exhibited excellent performance in discriminating between patients with lung cancer and healthy individuals. The lung cancer detection performance of the model achieved a sensitivity of 70.0% at 98% specificity in the test set. Notably, this model achieved a high sensitivity of 87.5% at 98% specificity level for SCC. And as a result of the LOD test, detection was possible even at tumor fractions as low as 0.1%.

Two primary approaches are commonly employed in methylation-based analyses: beta value and alpha value assessments^32^. The beta value quantifies methylation at the CpG site level and the alpha value quantifies methylation at the read level. Recent research has seen the development of algorithms that utilize α-values to predict tumor fractions^32,33^. Additionally, studies have been conducted to create models using fragment size and methylation information as separate features and an ensemble approach has been employed^34–36^. However, a method integrating fragment size and methylation information has not been studied previously. In this study, we introduced new features by capitalizing on the characteristics of ctDNA. We examined the association between fragment size and methylation. Shorter fragments were methylated in hypermethylated regions and unmethylated in hypomethylated regions. By combining these two ctDNA features, the AUC of cancer diagnostic models has been enhanced.

Algorithms that predict tumor fraction using methylation information have also been studied^32,33^. For CancerDetector, tumor cfDNA with a 1% tumor fraction was detected at low sequencing coverage (2×), and higher sequencing coverage (5× and 10×) improved the detection limit to 0.3%^32^. Because DISMIR was developed with a focus on binary classification problems, it is limited in predicting small tumor sizes^33^. In our study, the MFS model demonstrated a high level of sensitivity, detecting tumor fractions as low as 0.1%.

This study had several limitations. First, the sample size used in this study was relatively small. Therefore, it may be difficult to generalize this model. Validation of the model using additional samples is required. And the sample size in WGEM-seq data is insufficient to select DMRs. However, we confirmed that the methylation markers selected based on this sample exhibited reproducible differences between normal and cancer samples in the targeted EM-seq panel. Secondly, the LOD test should be conducted using multiple samples. However, each one sample from a patient with lung cancer and a healthy individual was used in this study, which could lead to biased results. Therefore, validation using additional data is required. Despite these limitations, we successfully established a noninvasive cfDNA methylation model for lung cancer detection.

This model incorporates methylation and fragment size information to enhance sensitivity. This method was validated in independent dataset, confirming its potential as a valuable tool for lung cancer detection.

### Supplementary Information

Supplementary Information.

## Data Availability

The MeDIP-seq, WGEM-seq, and Twist Methylome panel data were deposited in the Korea BioData Station (K-BDS, https://kbds.re.kr) with the accession ID, KAP240715. The targeted EM-seq panel data was deposited in Korean Nucleotide Archive (KoNA, https://kobic.re.kr/kona) with the accession ID, KAP230731. The 450K array data of tumor tissue and adjacent normal tissue samples used in this study were collected from The University of California Santa Cruz (UCSC) Xena database (https://xenabrowser.net/datapages/). The 450K array data of normal blood samples were collected from Gene Expression Omnibus (GEO) databases (https://www.ncbi.nlm.nih.gov/geo/).

## Code availability

The script for model evaluation can be accessed at https://github.com/minjung826/MFS_model.
