# Supplementary material for: Deep learning model integrating cfDNA methylation and fragment size profiles for lung cancer diagnosis
Source: Sci Rep. 2024 Jun 26;14:14797. doi: 10.1038/s41598-024-63411-2 (PMC11208569; doi:10.1038/s41598-024-63411-2)
Supplement: Supplementary file 1 — Supplementary Information. [file 41598_2024_63411_MOESM1_ESM.docx]

**Supplementary Figures, Tables**

**Supplementary Figure 1.** Example of MFS table calculation.


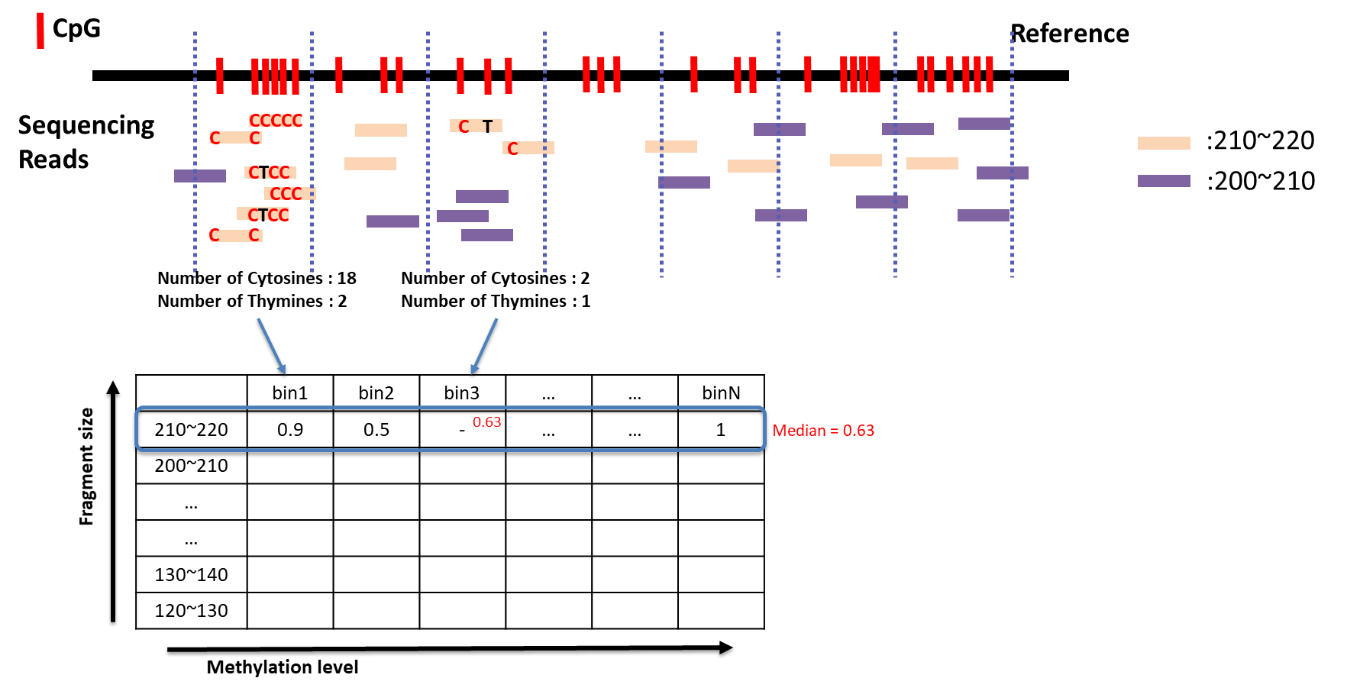


**Supplementary Figure 2.** Lung cancer methylation marker selection workflow. Tumor specific methylation markers were selected using 450K array, WGEM-seq and MeDIP-seq data, and marker filtering was performed using Twist Human Methylome Panel data.


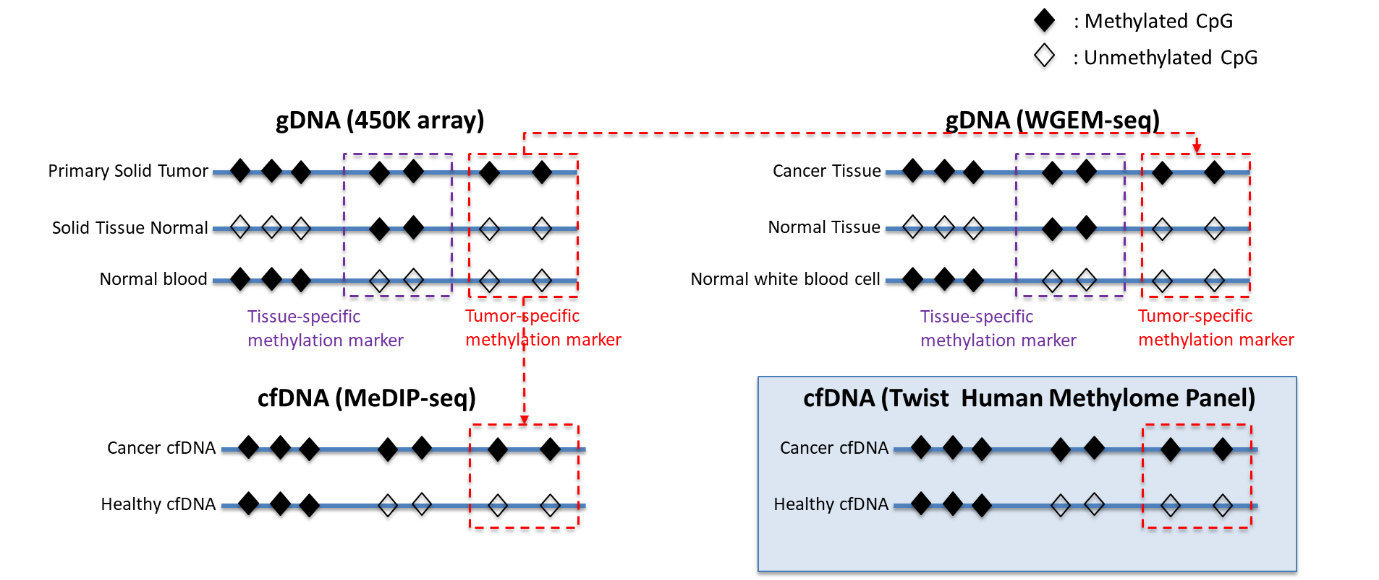


**Supplementary Figure 3.** Differentially methylated regions between lung cancer and normal samples. a. t-SNE plot of selected markers from the 450K array and MeDIP-seq. b. t-SNE plot of selected markers from the 450K array and WGEM-seq.

a


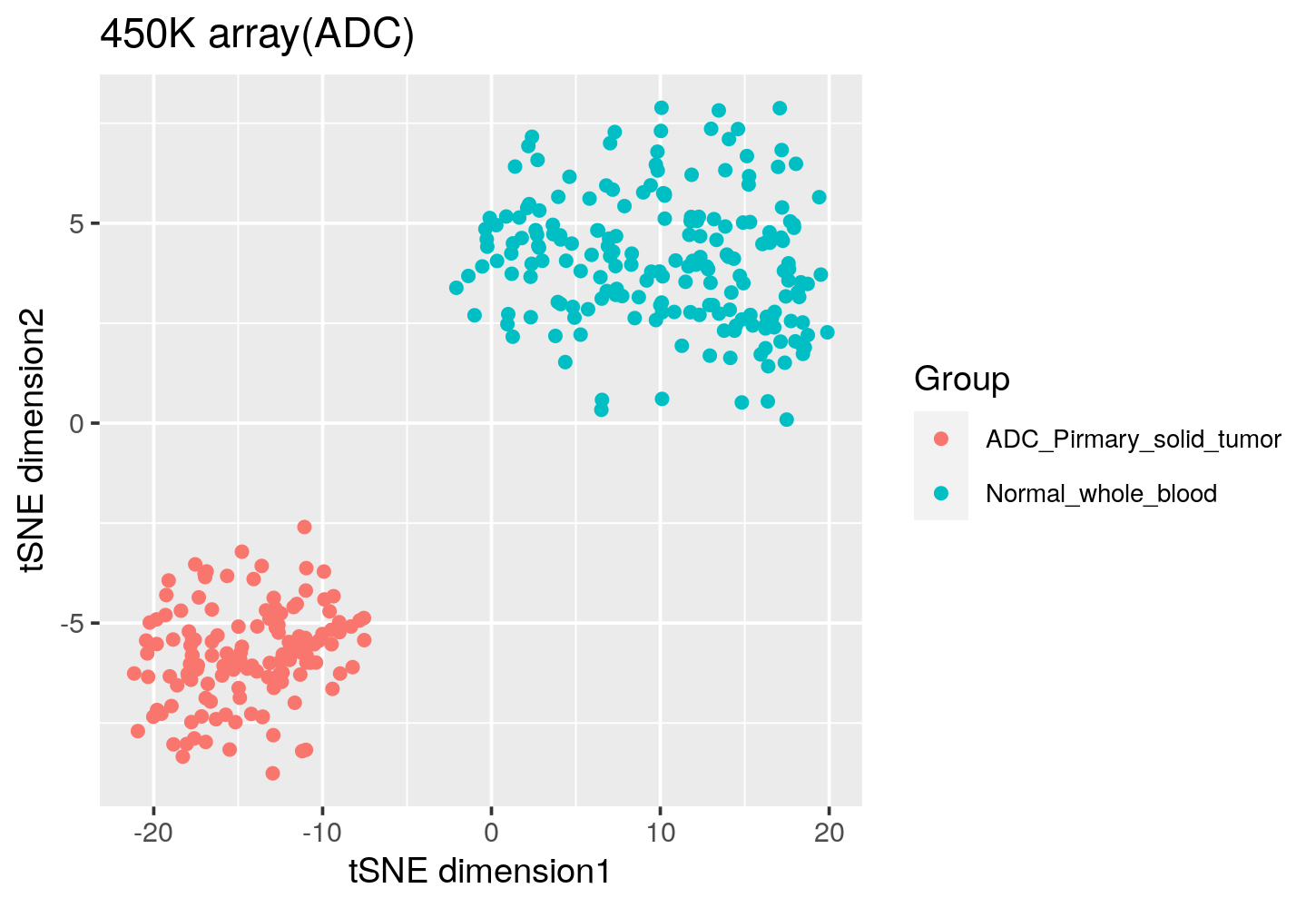

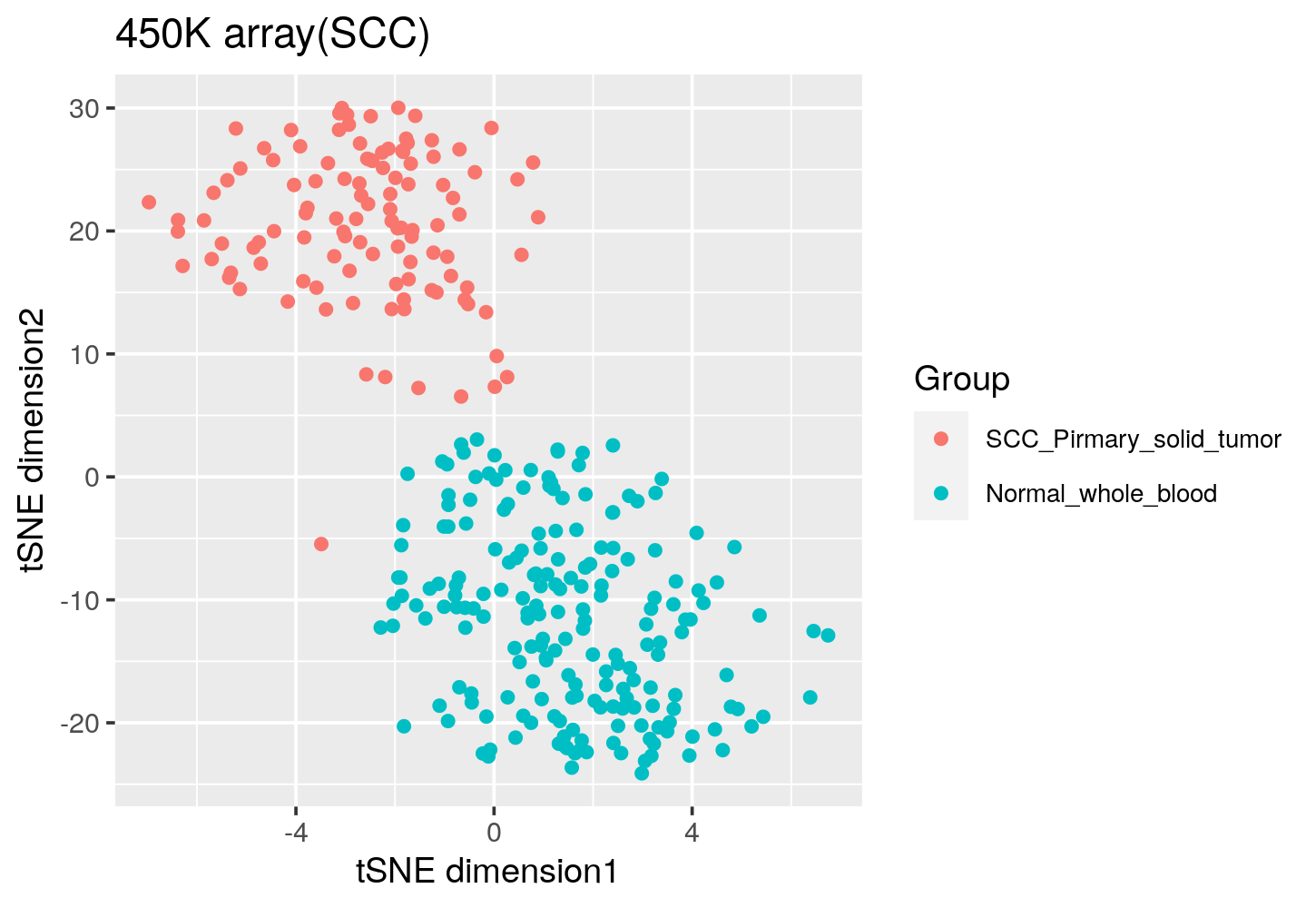

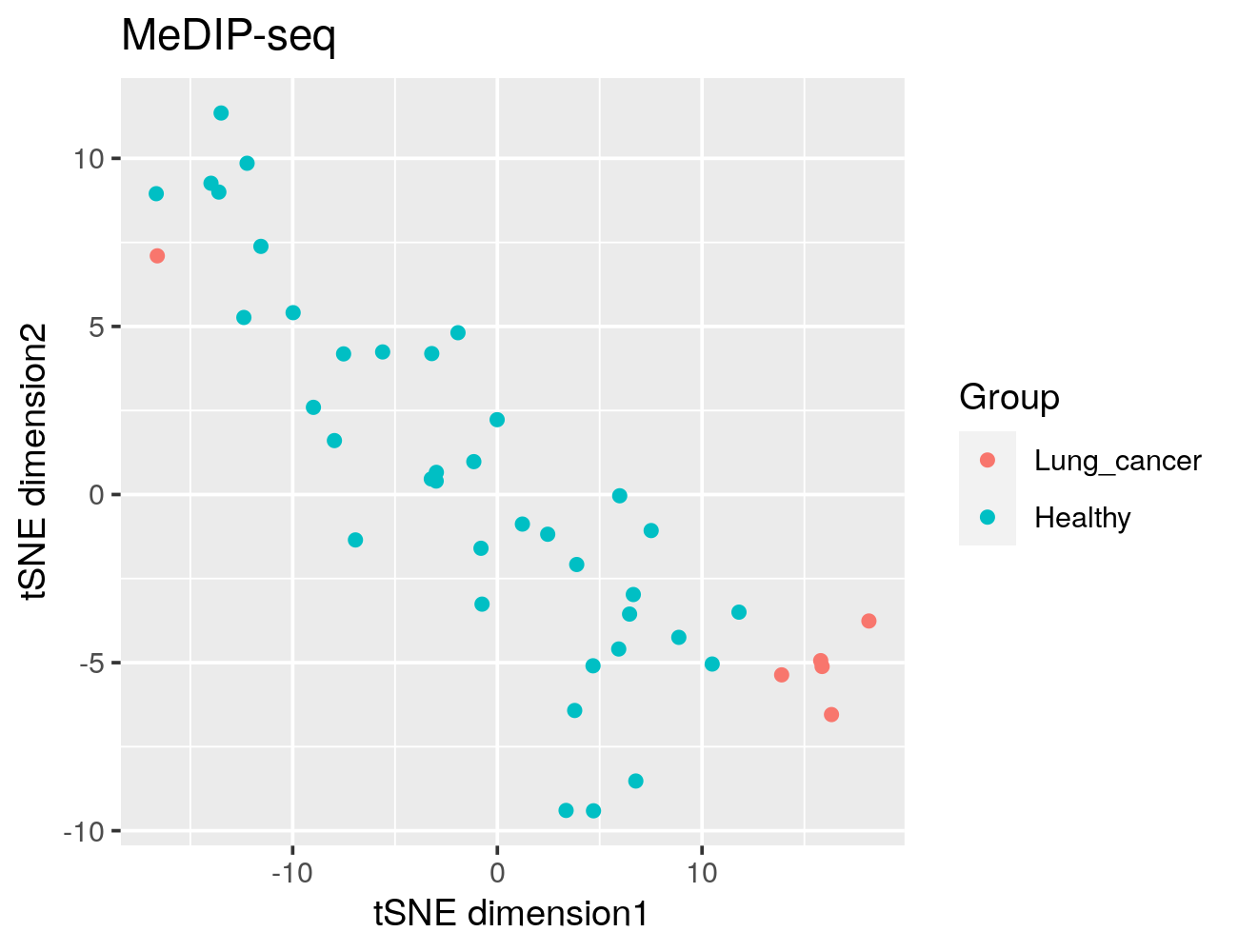


b


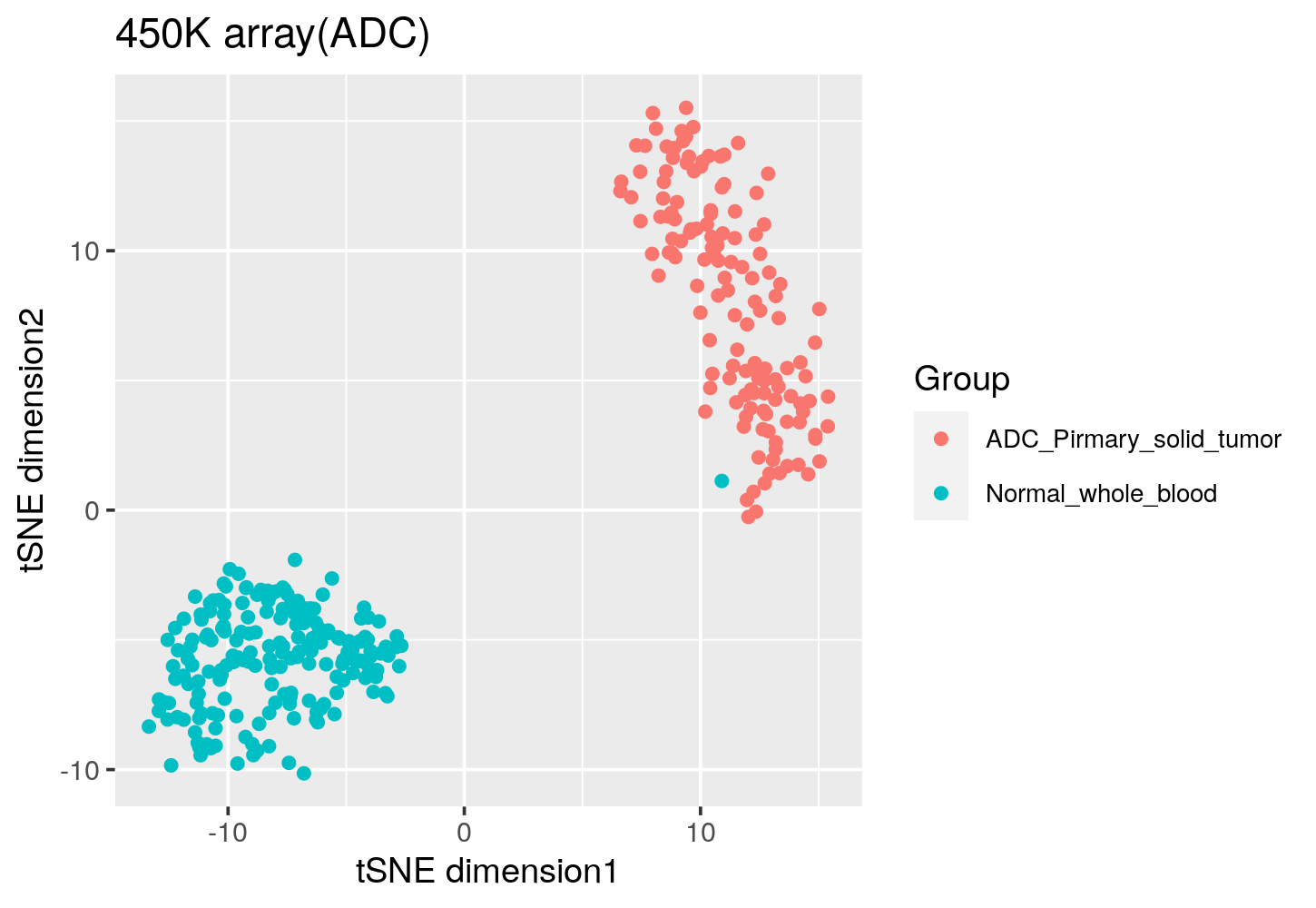

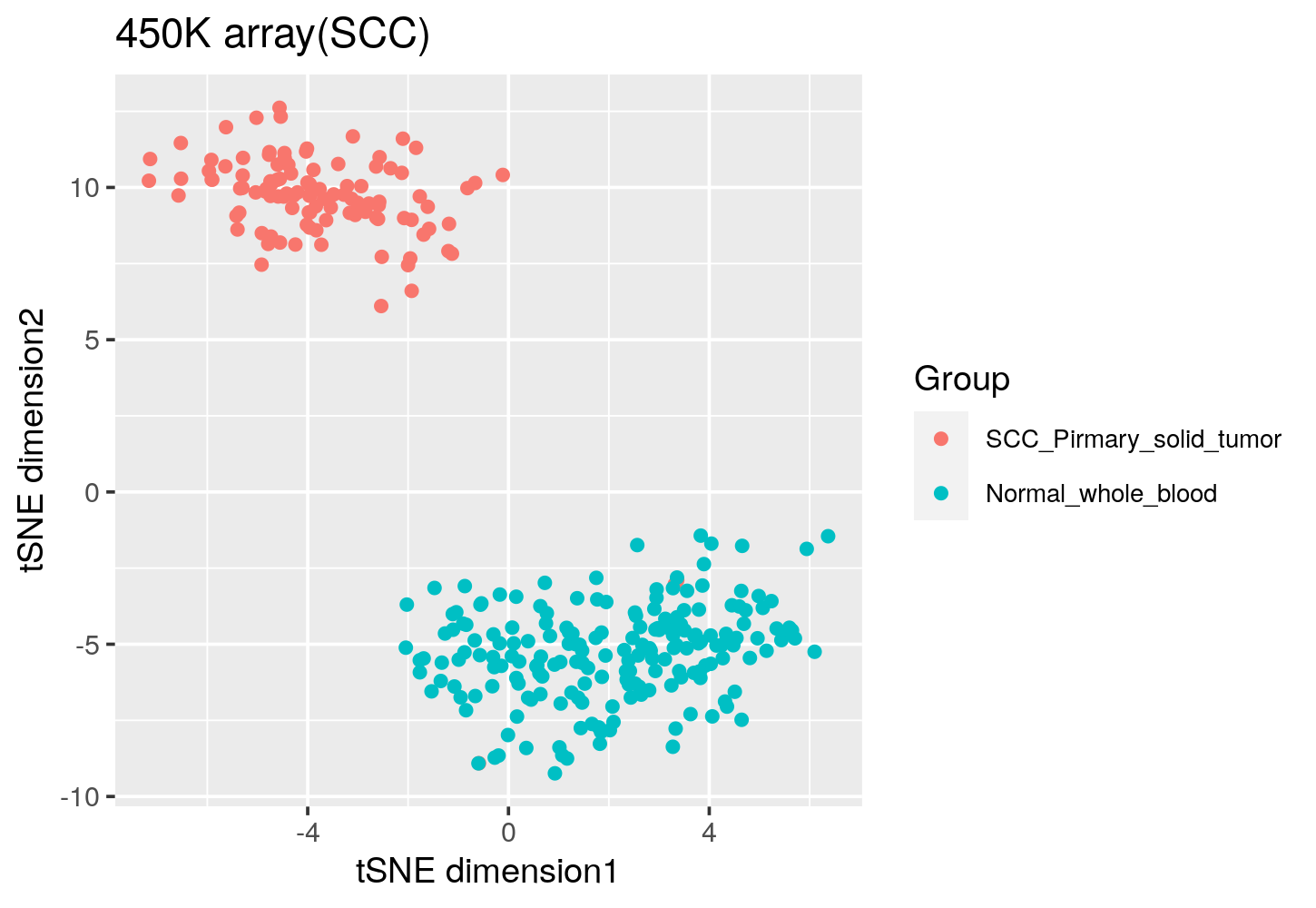

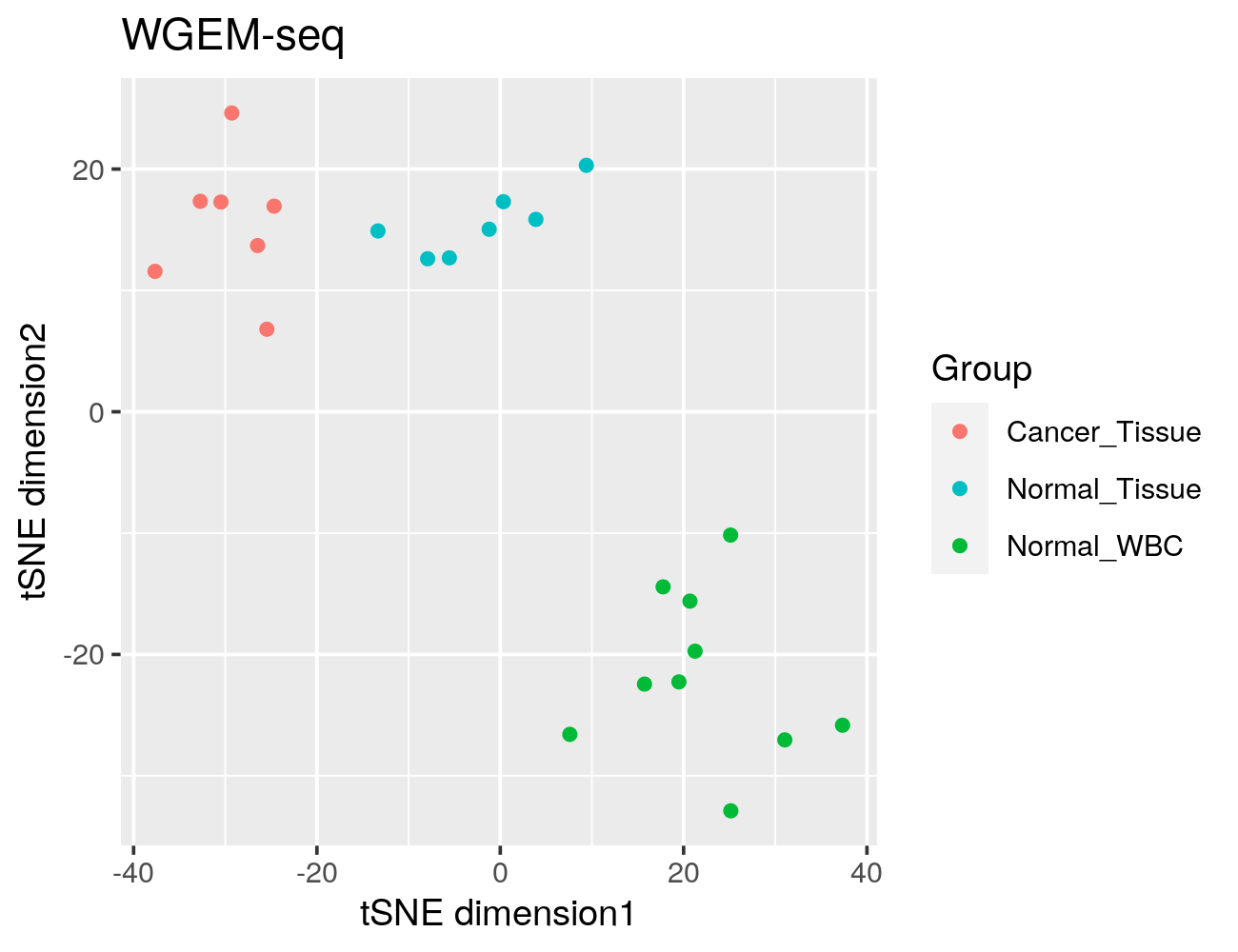


**Supplementary Figure 4.** Subtype-specific differentially methylated regions and common differentially methylated regions of ADC and SCC samples. a. Hypermethylated regions. b. Hypomethylated regions.

a


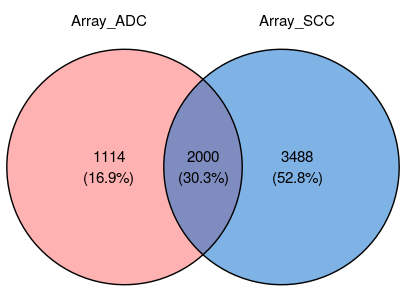


b


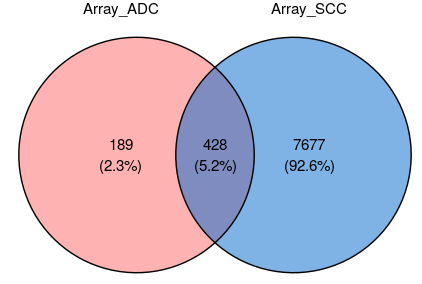


**Supplementary Figure 5.** Integrated differentially methylated regions. a. Hypermethylated regions. b. Hypomethylated regions.

a


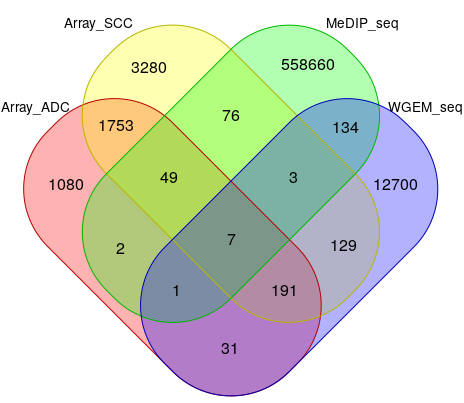


b


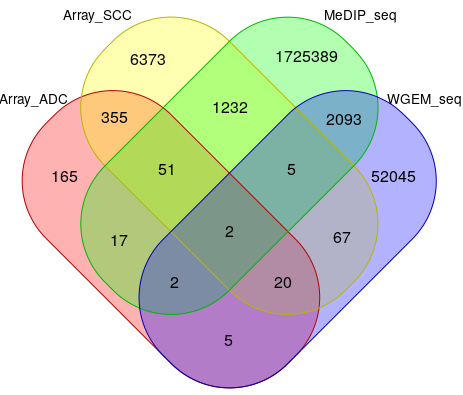


**Supplementary Figure 6.** Significant methylated markers between patients with lung cancer and healthy individuals. a. t-SNE plot on Twist Human Methylome panel. b. heatmap of hypermethylated and hypomethylated markers on Twist Human Methylome panel.

a


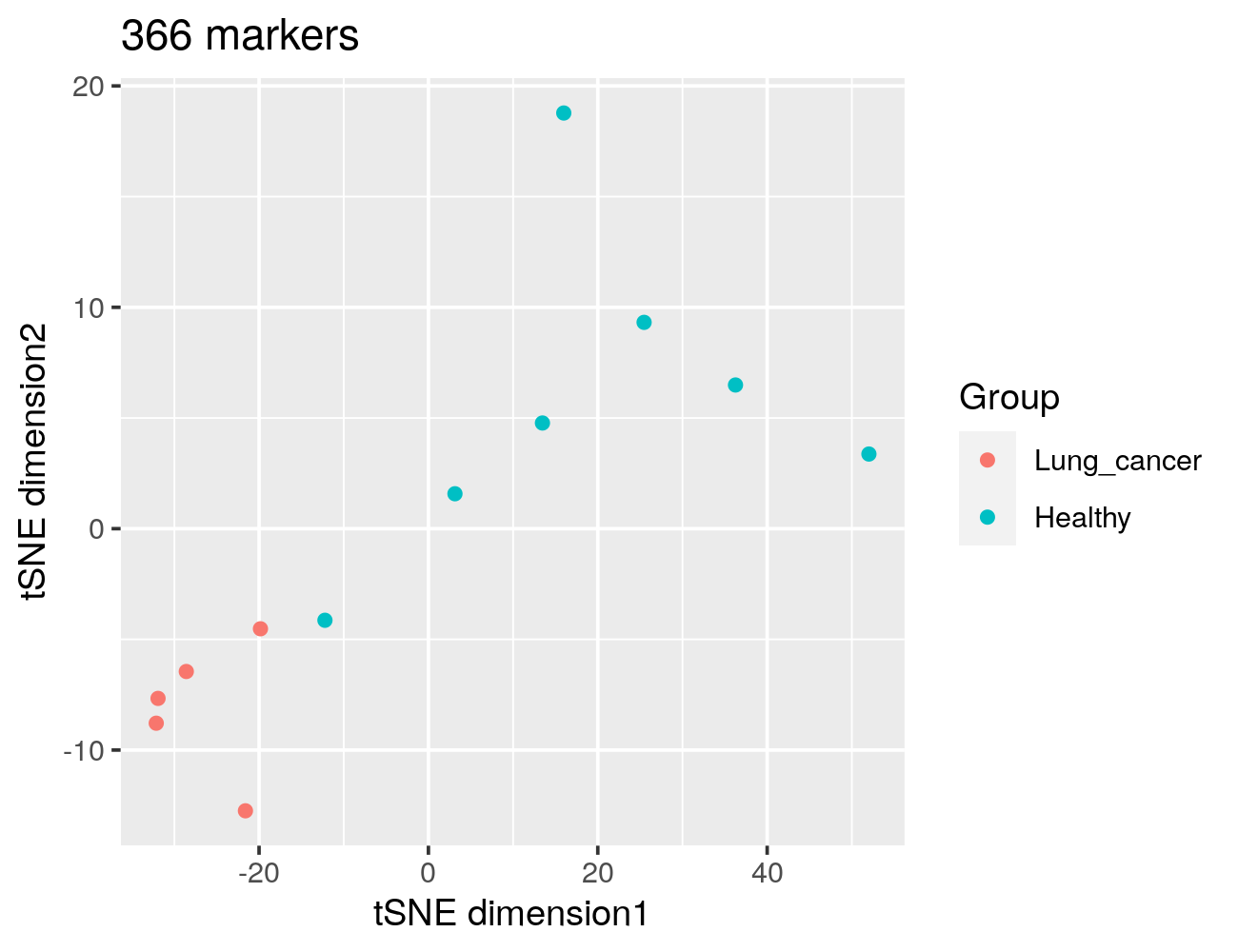


b


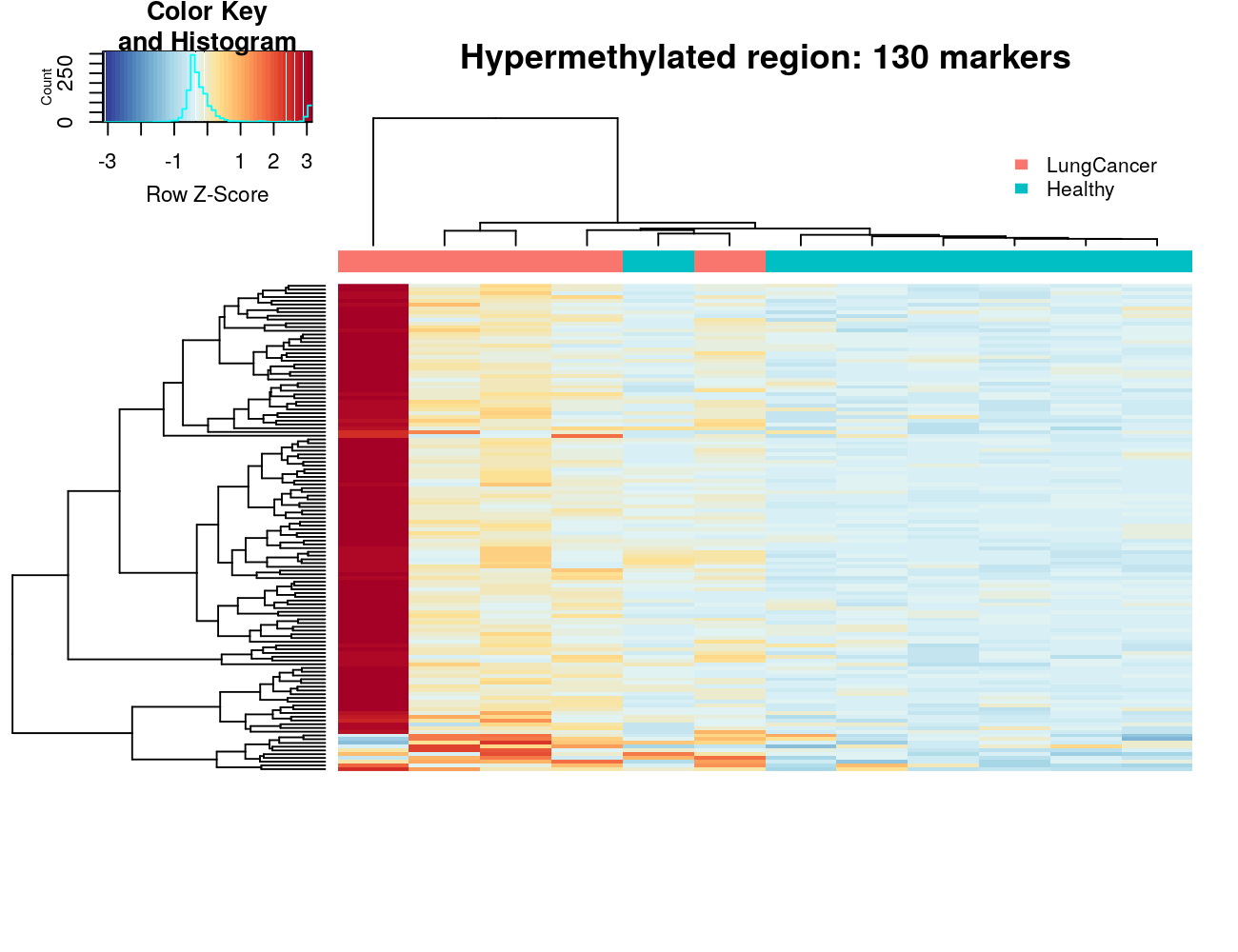

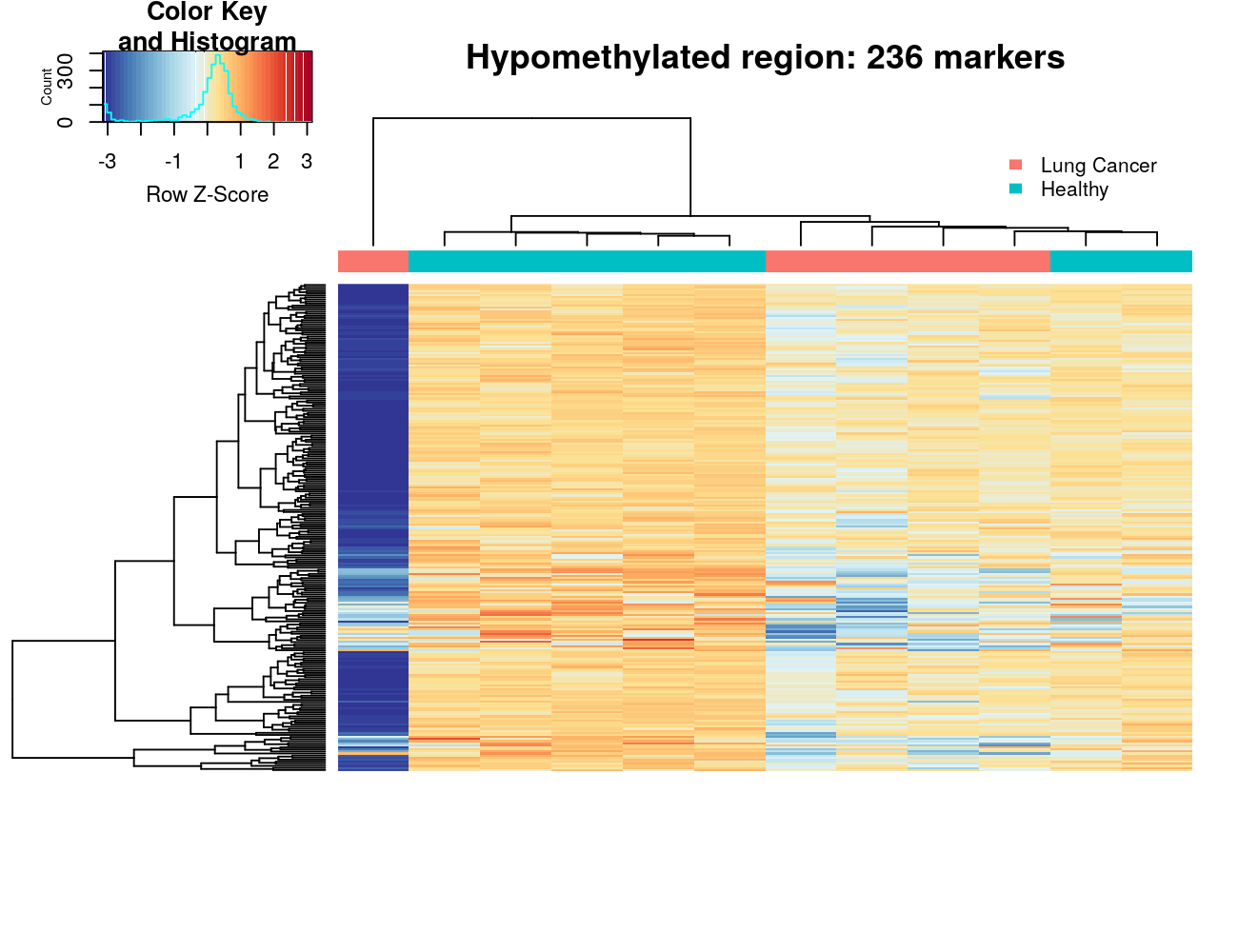


**Supplementary Figure 7.** Functional annotation. a. Hypermethylated regions b. Hypomethylated regions.

a


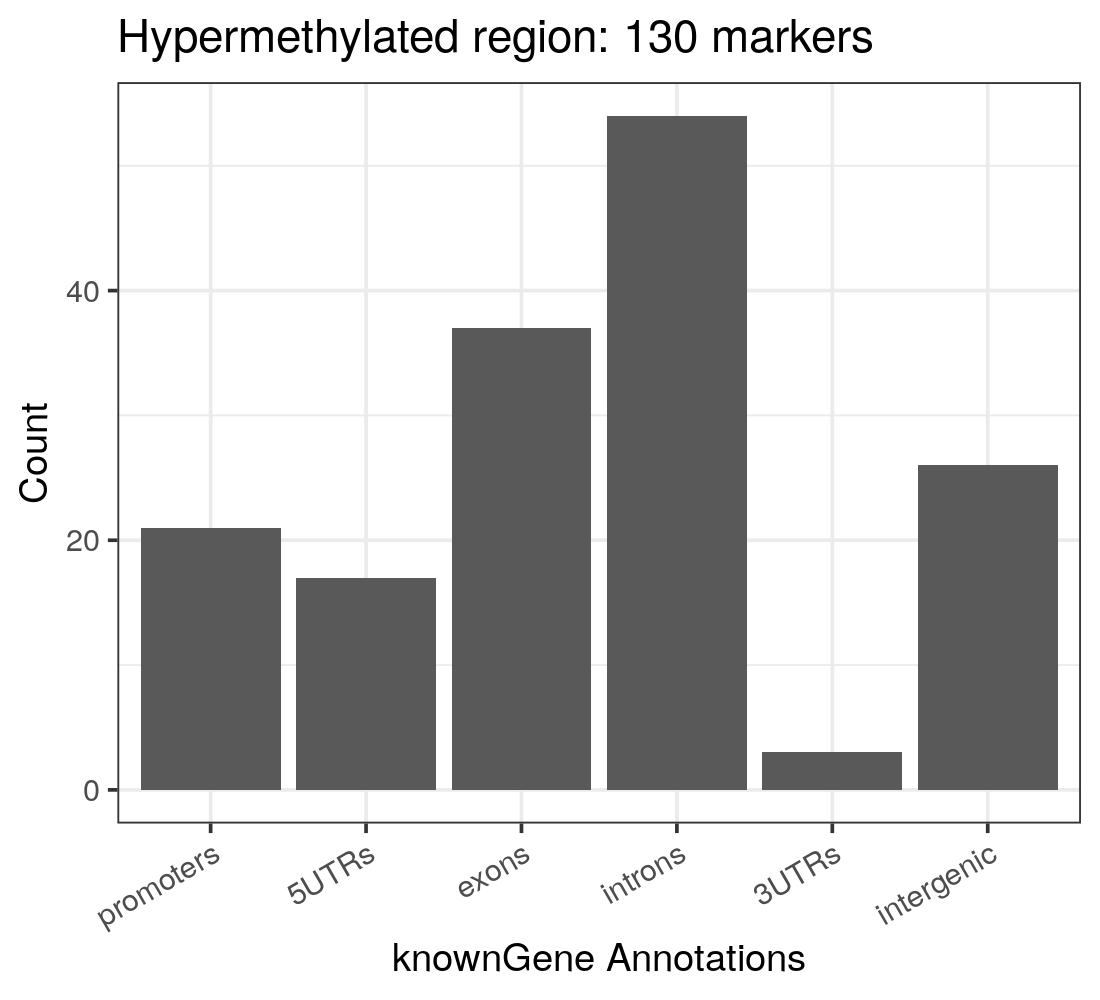

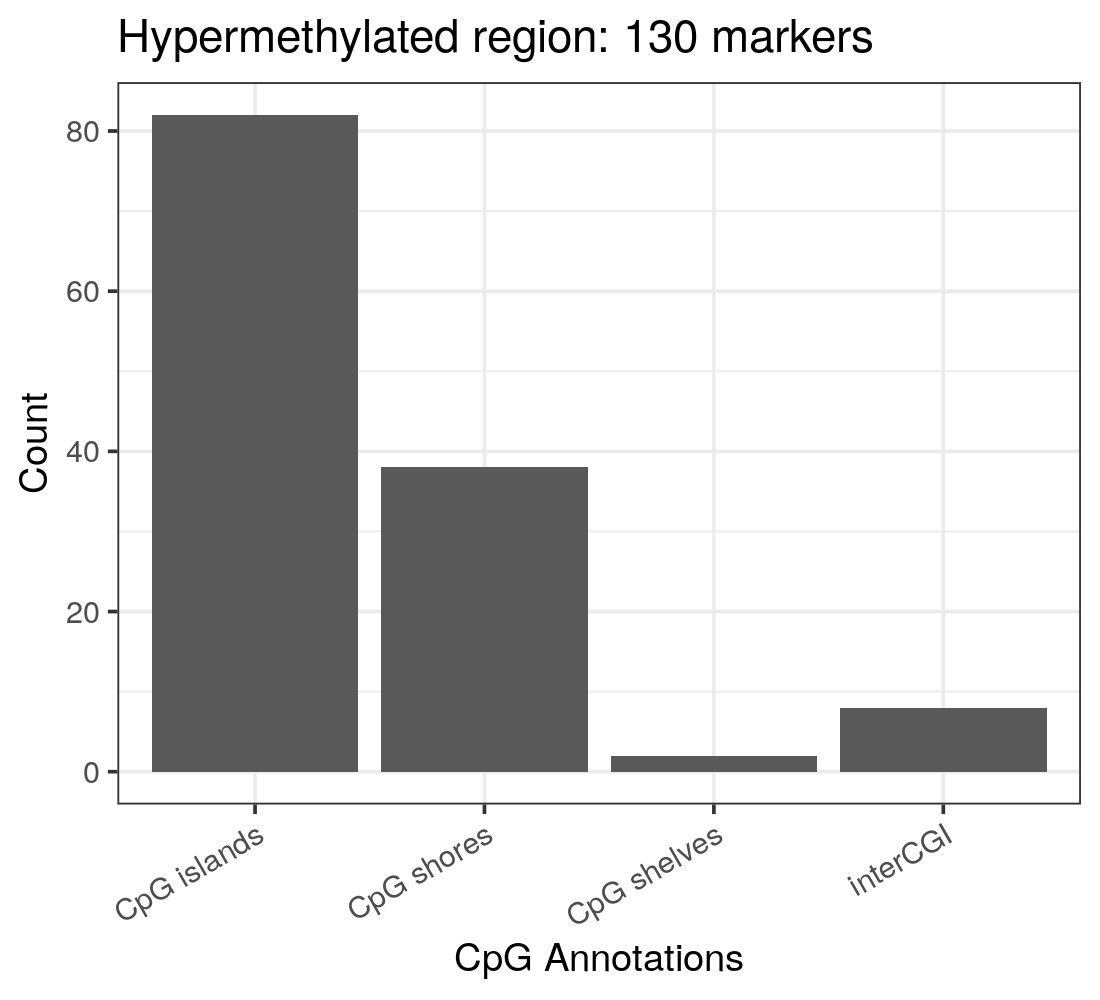


b


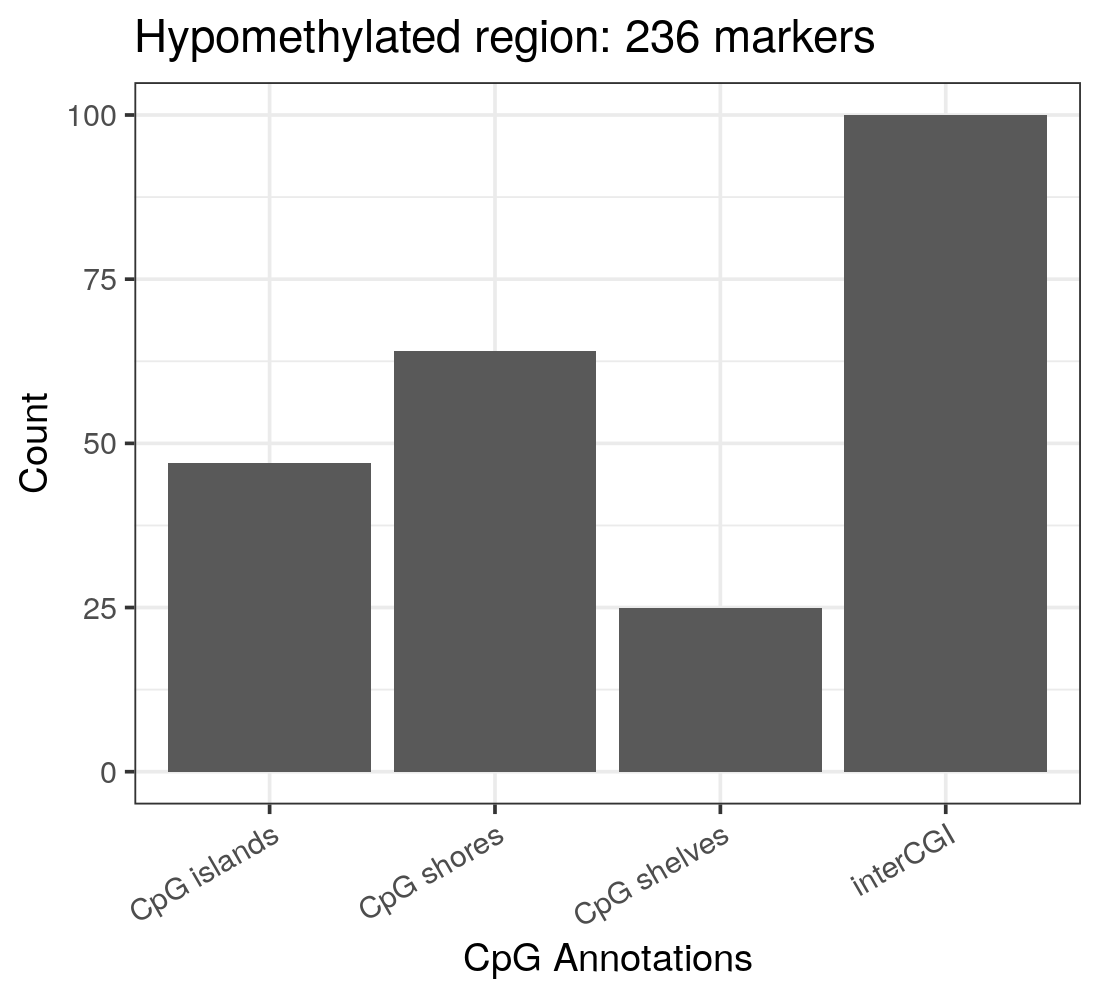


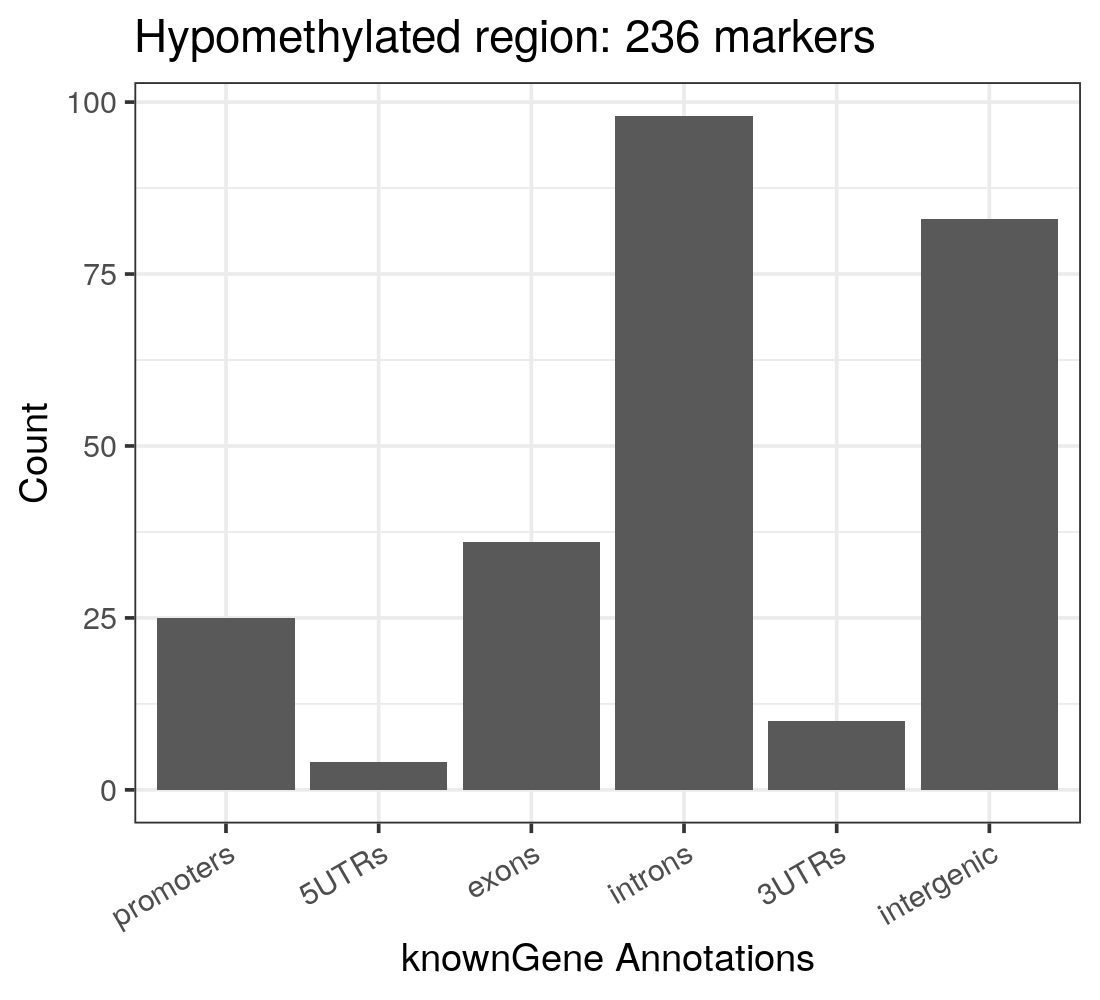


**Supplementary Figure 8.** Noise removal on targeted EM-seq panel. Beta value before and after noise removal.


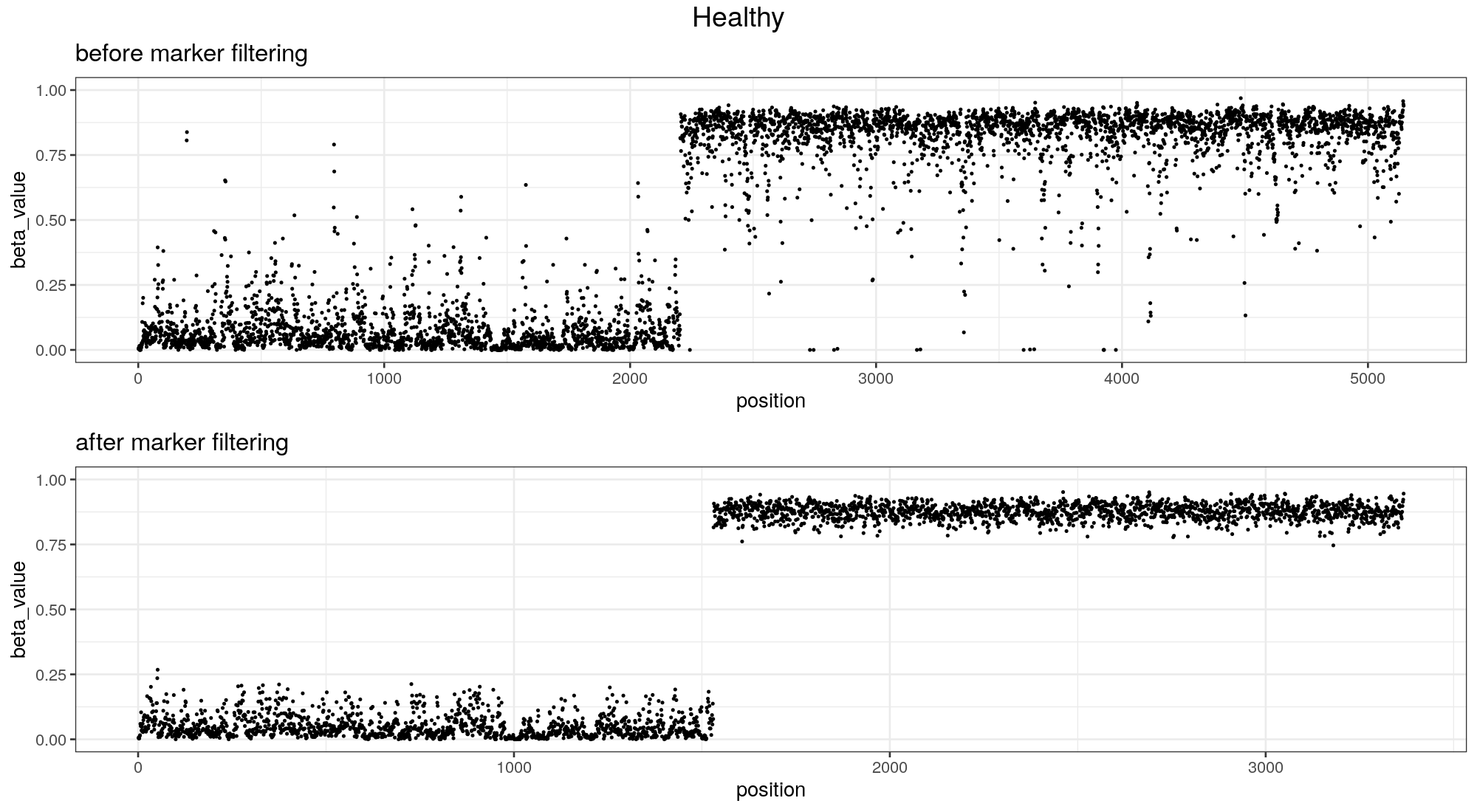


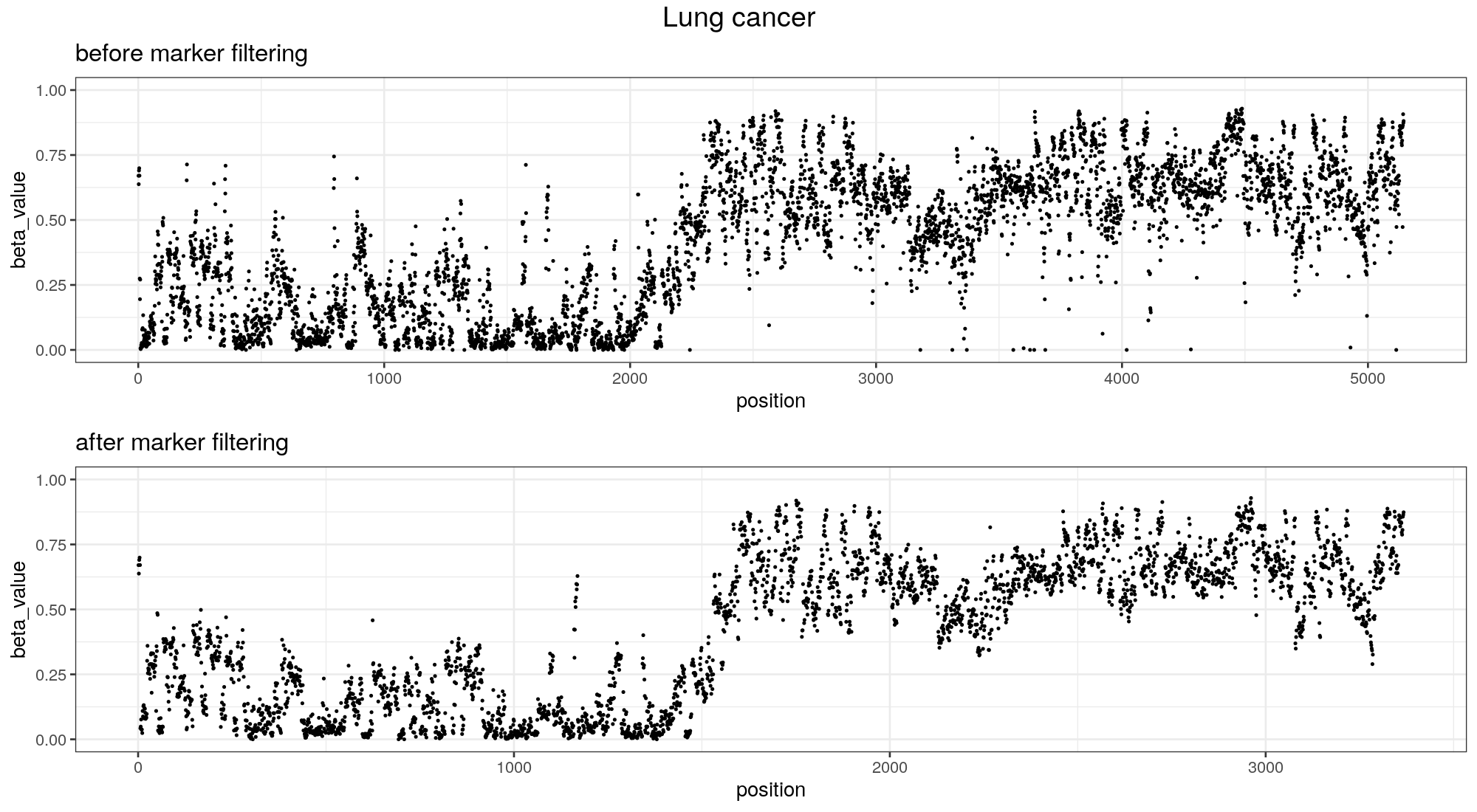


**Supplementary Figure 9.** Sensitivity at 95% specificity by dataset.


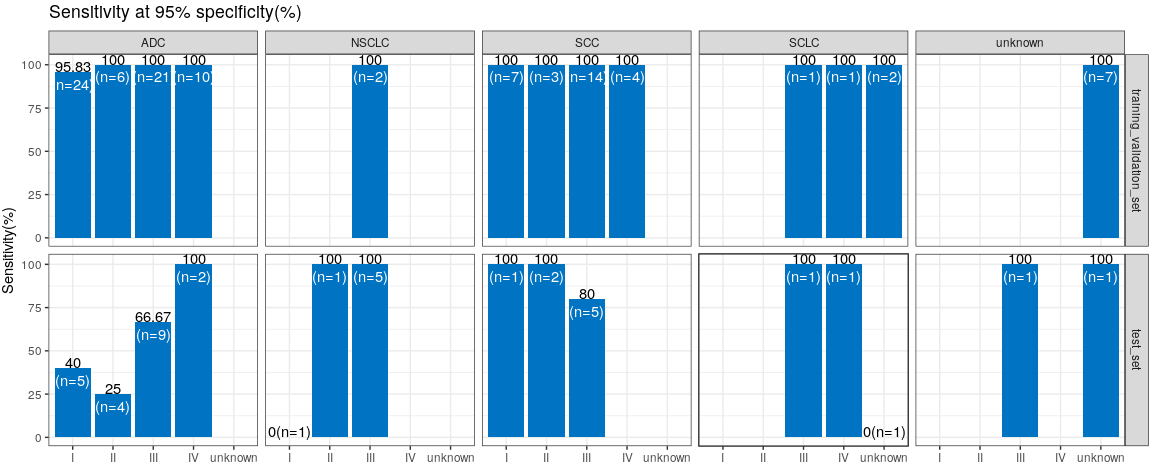


**Supplementary Table 1.** Clinical data in MeDIP-seq

|  | Lung cancer | Healthy individuals |
| --- | --- | --- |
| Total (n) | 25 | 190 |
| Age (years) |  |  |
| Mean | 66 | 32 |
| Range | 45-83 | 23-53 |
| Gender |  |  |
| Male, n(%) | 19 (76) | 78 (41.05) |
| Female, n(%) | 6 (24) | 112 (58.95) |
| Stage |  |  |
| Ⅰ, n(%) | 7 (28) | - |
| Ⅱ, n(%) | 5 (20) | - |
| Ⅲ, n(%) | 13 (52) | - |
| Ⅳ, n(%) | 0 (0) | - |
| unknown, n(%) | 0 (0) | - |
| Subtype |  |  |
| ADC, n(%) | 17 (68) | - |
| NSCLC-NOS, n(%) | 0 (0) | - |
| SCC, n(%) | 7 (28) | - |
| SCLC, n(%) | 1 (4) | - |
| unknown, n(%) | 0 (0) | - |

**Supplementary Table 2.** Clinical data in WGEM-seq

|  | Lung cancer | Healthy individuals |
| --- | --- | --- |
| Total (n) | 7 | 10 |
| Age (years) |  |  |
| Mean | 68 | 32 |
| Range | 50-78 | 23-52 |
| Gender |  |  |
| Male, n(%) | 3 (42.86) | 5 (50) |
| Female, n(%) | 4 (57.14) | 5 (50) |
| Stage |  |  |
| Ⅰ, n(%) | 5 (71.43) | - |
| Ⅱ, n(%) | 2 (28.57) | - |
| Ⅲ, n(%) | 0 (0) | - |
| Ⅳ, n(%) | 0 (0) | - |
| unknown, n(%) | 0 (0) | - |
| Subtype |  |  |
| ADC, n(%) | 6 (85.71) | - |
| NSCLC-NOS, n(%) | 0 (0) | - |
| SCC, n(%) | 1 (14.29) | - |
| SCLC, n(%) | 0 (0) | - |
| unknown, n(%) | 0 (0) | - |

**Supplementary Table 3.** Clinical data in Twist Human Methylome panel

|  | Lung cancer | Healthy individuals |
| --- | --- | --- |
| Total (n) | 5 | 7 |
| Age (years) |  |  |
| Mean | 60 | 32 |
| Range | 45-73 | 24-49 |
| Gender |  |  |
| Male, n(%) | 4 (80) | 2 (28.57) |
| Female, n(%) | 1 (20) | 5 (71.43) |
| Stage |  |  |
| Ⅰ, n(%) | 1 (20) | - |
| Ⅱ, n(%) | 1 (20) | - |
| Ⅲ, n(%) | 3 (60) | - |
| Ⅳ, n(%) | 0 (0) | - |
| unknown, n(%) | 0 (0) | - |
| Subtype |  |  |
| ADC, n(%) | 2 (40) | - |
| NSCLC-NOS, n(%) | 0 (0) | - |
| SCC, n(%) | 2 (40) | - |
| SCLC, n(%) | 1 (20) | - |
| unknown, n(%) | 0 (0) | - |

**Supplementary Table 4.** Composition of each dataset for marker selection. a. 450K array of ADC. b. 450K array of SCC. c. MeDIP-seq. d. WGEM-seq. e. Twist Human Methylome Panel

a

| Type | Cancer tissue | Normal tissue | Normal blood | Total |
| --- | --- | --- | --- | --- |
| Discovery set | 320 | 32 | 459 | 811 |
| Validation set | 138 | 0 | 197 | 335 |
| Total | 458 | 32 | 656 | 1,146 |

b

| Type | Cancer tissue | Normal tissue | Normal blood | Total |
| --- | --- | --- | --- | --- |
| Discovery set | 259 | 42 | 459 | 760 |
| Validation set | 111 | 0 | 197 | 308 |
| Total | 370 | 42 | 656 | 1,068 |

c

| Type | Cancer | Normal | Total |
| --- | --- | --- | --- |
| Discovery set | 19 | 152 | 171 |
| Validation set | 6 | 38 | 44 |
| Total | 25 | 190 | 215 |

d

| Type | Cancer tissue | Normal tissue | WBC | Total |
| --- | --- | --- | --- | --- |
| Discovery set | 7 | 7 | 10 | 24 |

WBC, white blood cells;

e

| Type | Cancer | Normal | Total |
| --- | --- | --- | --- |
| Discovery set | 5 | 7 | 12 |

| Type | Data | Hypermethylated markers | Hypomethylated markers |
| --- | --- | --- | --- |
| 450K array ∩ MeDIP-seq | n=1112 (579 lung cancer VS 533 normal)  n=171 (19 lung cancer VS 152 normal) | 138 | 1,309 |
| 450K array ∩ WGEM-seq | n=1112 (579 lung cancer VS 533 normal)  n=24 (7 lung cancer VS 17 normal) | 362 | 101 |

**Supplementary Table 5.** Lung cancer specific methylation markers selected from each dataset.

| Subtype | Data | Hypermethylated markers | Hypomethylated markers |
| --- | --- | --- | --- |
| ADC | n=811 (320 lung cancer_ADC VS 491 normal) | 3,114 | 617 |
| SCC | n=760 (259 lung cancer_SCC VS 501 normal) | 5,488 | 8,105 |

**Supplementary Table 6.** Subtype specific methylation markers.
